# Correction: Age trends in asymptomatic and symptomatic *Leishmania donovani* infection in the Indian subcontinent: A review and analysis of data from diagnostic and epidemiological studies

**DOI:** 10.1371/journal.pntd.0007150

**Published:** 2019-02-26

**Authors:** The PLOS Neglected Tropical Diseases Staff

## Notice of Republication

This article was republished on December 14, 2018, to correct errors in the Supporting Files that were introduced during the typesetting process. The publisher apologizes for the errors. Please download this article again to view the correct version. The originally published, uncorrected article and the republished, corrected articles are provided here for reference.

## Supporting information

S1 File Originally published, uncorrected article. (PDF)

S2 File Republished, corrected article. (PDF)
