# Supplementary material for: Age trends in asymptomatic and symptomatic Leishmania donovani infection in the Indian subcontinent: A review and analysis of data from diagnostic and epidemiological studies
Source: PLoS Negl Trop Dis. 2018 Dec 6;12(12):e0006803. doi: 10.1371/journal.pntd.0006803 (PMC6283524; doi:10.1371/journal.pntd.0006803)
Supplement: S1 Text — (DOCX) [file pntd.0006803.s002.docx]

**Details of literature search**

**Database:** PubMed (<https://www.ncbi.nlm.nih.gov/pubmed/>)

**Search Terms:** (visceral leishmaniasis[Title/Abstract] OR kala-azar[Title/Abstract] OR leishmania [Title/Abstract]) AND (age[Title/Abstract] OR asymptomatic[Title/Abstract] OR epidemiology[Title/Abstract] OR epidemiological[Title/Abstract] OR immunology[Title/Abstract] OR immunological[Title/Abstract] OR serology[Title/Abstract] OR serological[Title/Abstract] OR seroepidemiological[Title/Abstract] OR sero-epidemiological[Title/Abstract] OR seropositive[Title/Abstract] OR seroprevalence[Title/Abstract] OR sero-positive[Title/Abstract] OR sero-prevalence[Title/Abstract] OR sero-survey[Title/Abstract] OR direct agglutination test[Title/Abstract] OR DAT[Title/Abstract] OR rK39[Title/Abstract] OR K39[Title/Abstract] OR rK28[Title/Abstract] OR recombinant K39 antigen[Title/Abstract] OR Montenegro test[Title/Abstract] OR leishmanin skin test[Title/Abstract] OR LST[Title/Abstract] OR interferon gamma[Title/Abstract] OR IGRA[Title/Abstract] OR interferon gamma release assay[Title/Abstract] OR ELISA[Title/Abstract] OR rapid diagnostic test[Title/Abstract] OR urine[Title/Abstract] OR polymerase chain reaction[Title/Abstract] OR qPCR[Title/Abstract] OR parasite DNA[Title/Abstract]) NOT (mucocutaneous leishmaniasis[Title/Abstract] OR cutaneous leishmaniasis[Title/Abstract] OR Brazil[Title/Abstract] OR South America[Title/Abstract]) AND (India[Title/Abstract] OR Indian[Title/Abstract] OR Bangladesh[Title/Abstract] OR Bangladeshi[Title/Abstract] OR Nepal[Title/Abstract] OR Nepalese[Title/Abstract] OR South East Asia[Title/Abstract] OR Thailand[Title/Abstract] OR Bhutan[Title/Abstract])

**Date searched:** 8/10/2017

**Yield:** 374 articles

**Potentially relevant articles identified from 5 reviews**

| **Review** | **Number of references** |
| --- | --- |
| Bern et al, 2010 [1] | 96 |
| Cameron et al, 2016 [2] | 52 |
| Guerin et al, 2002 [3] | 76 |
| Hirve et al, 2016 [4] | 175 |
| Soni et al, 2013 [5] | 40 |
| **Total** | **439** |

**References**

1. Bern C, Courtenay O, Alvar J. Of Cattle, Sand Flies and Men: A Systematic Review of Risk Factor Analyses for South Asian Visceral Leishmaniasis and Implications for Elimination. PLoS Negl Trop Dis. 2010;4(2):e599.

2. Cameron MM, Acosta-Serrano A, Bern C, Boelaert M, Den Boer M, Burza S, et al. Understanding the transmission dynamics of Leishmania donovani to provide robust evidence for interventions to eliminate visceral leishmaniasis in Bihar, India. Parasites and Vectors. 2016;9(1).

3. Guerin PJ, Olliaro P, Sundar S, Boelaert M, Croft SL, Desjeux P, et al. Visceral leishmaniasis: current status of control, diagnosis, and treatment, and a proposed research and development agenda. 2002;2:494–501.

4. Hirve S, Boelaert M, Matlashewski G, Mondal D, Arana B, Kroeger A, et al. Transmission Dynamics of Visceral Leishmaniasis in the Indian Subcontinent – A Systematic Literature Review. PLoS Negl Trop Dis. 2016;10(8):e0004896. Available from: http://dx.plos.org/10.1371/journal.pntd.0004896

5. Soni P, Mishra R, Kumar G, Pandey P, Kumar P. Kala-Azar Elimination in Bihar : Adoption of Newer Evidence-based Strategies Required for Elimination. Middle-East J Sci Res. 2013;17(9):1220–7.
