# Supplementary material for: Age trends in asymptomatic and symptomatic Leishmania donovani infection in the Indian subcontinent: A review and analysis of data from diagnostic and epidemiological studies
Source: PLoS Negl Trop Dis. 2018 Dec 6;12(12):e0006803. doi: 10.1371/journal.pntd.0006803 (PMC6283524; doi:10.1371/journal.pntd.0006803)
Supplement: S2 Text — Model fitting and comparison, and parameter estimates. (PDF) [file pntd.0006803.s003.pdf]

Age trends in asymptomatic and symptomatic Leishmania donovani infection in the Indian subcontinent: a review and analysis of data from diagnostic and epidemiological studies - S2 Text

Lloyd A.C. Chapman, Alex L.K. Morgan, Emily R. Adams, Caryn Bern, Graham F. Medley, T. Déirdre Hollingsworth

Contents

1 Methods **1**
1.1 Reversible catalytic model . . . 1
1.2 Parameter estimation . . . 2
1.3 Model comparison . . . 3

2 Results **3**
2.1 Hasker et al (2013) data . . . 3
2.2 Model selection . . . 4
2.3 Parameter estimates . . . 4

1 Methods

1.1 Reversible catalytic model

The general form of the reversible catalytic model fitted to the data is as follows. The transmission dynamics are assumed to be in equilibrium, such that the prevalence of infection (as determined by sero-/LST-/PCR-positivity) only varies with age a and not time. Sero-/LST-/PCR-negative individuals, whose prevalence is denoted by s, become sero-/LST-/PCR-positive (prevalence p) at a certain, potentially study-, test- and age-dependent, rate $\lambda_i(a)$ (which we refer to as the rate of infection (ROI)), and revert to sero-/LST-/PCR-negativity at an age-independent, but potentially study- and test-dependent, rate γ_i, where $i \in \{1, \dots, N_s\}$ or $i \in \{\mathrm{DAT}, \mathrm{rK39}, \mathrm{LST}, \mathrm{PCR}\}$ and N_s is the number of studies, i.e.

$$\frac{\mathrm{d}s}{\mathrm{d}a} = -\lambda_i(a)s + \gamma_i p, \tag{1}$$

$$\frac{\mathrm{d}p}{\mathrm{d}a} = \lambda_i(a)s - \gamma_i p, \tag{2}$$

$$p(0) = 0. \tag{3}$$

Equations (1)–(3) can be reduced to an initial value problem for p since $s + p = 1$,

$$\frac{\mathrm{d}p}{\mathrm{d}a} = \lambda_i(a)(1-p) - \gamma_i p, \quad \text{s.t.} \quad p(0) = 0. \tag{4}$$

Data from Hasker et al [1] suggests that seroconversion rate increases with age. To test whether the conversion rate is age-dependent we consider different forms of $\lambda(a)$:

- Constant (age-independent) ROI:

$$\lambda_i(a) = b_{0,i}. \tag{5}$$

For this form, the solution of (4) is

$$p(a) = \frac{b_{0,i}}{b_{0,i} + \gamma_i}(1 - e^{-(b_{0,i}+\gamma_i)a}). \tag{6}$$

- Age-dependent ROI:
  We assume that the rate of conversion to sero-/LST-/PCR-positivity increases linearly with age, based on the data in [1] (see below)
  $$\lambda_i(a) = b_{0,i} + b_{1,i}a, \tag{7}$$
  where $b_{1,i} \geq 0$ is the rate at which the conversion rate increases with age. The initial value problem for p does not have a simple closed form solution in this case.

Since the conversion and reversion rates may also vary with the location and time period in which the study was performed, and/or the test used, we compare the model fit under different assumptions about the study- and test-dependence as shown in Table 1. We note that the age-independent models are nested inside the age-dependent models (they are obtained by setting $b_{1,i} = 0$).

Table 1. Summary of the different age-independent and age-dependent models tested

Model	ROI, $\lambda_i(a)$	Reversion rate, γ_i
Age-independent, $\lambda_i(a) = b_{0,i}$ $(b_{1,i} = 0)$		
1	const.	const.
2	test-specific	const.
3	study-specific	const.
4	test-specific	test-specific
5	study-specific	test-specific
6	study-specific	study-specific
Age-dependent, $\lambda_i(a) = b_{0,i} + b_{1,i}a$		
1a	indep. of study/test	indep. of study/test
2a	test-specific	indep. of study/test
3a	study-specific	indep. of study/test
4a	test-specific	test-specific
5a	study-specific	test-specific
6a	study-specific	study-specific

$*$ ROI = Rate of infection

1.2 Parameter estimation

The catalytic model was fitted to the infection prevalence data from the studies in Table 3 of the main text to estimate the ROIs (the baseline ROIs, $\mathbf{b}_0 = (b_{0,i})_{i=1,\ldots,N_s}$, and rates of increase with age, $\mathbf{b}_1 = (b_{1,i})_{i=1,\ldots,N_s}$, where applicable) and reversion rates ($\boldsymbol{\gamma} = (\gamma_i)_{i=1,\ldots,N_s}$) using maximum likelihood estimation. The overall binomial likelihood is given by

$$L(\mathbf{b}_0, \mathbf{b}_1, \boldsymbol{\gamma}) = \prod_{i=1}^{N_s} \left(\prod_{j=1}^{N_i} p_{i,j}^{k_{i,j}} (1 - p_{i,j})^{n_{i,j} - k_{i,j}} \right), \tag{8}$$

where N_i is the number of age groups in study i, $p_{i,j} = p(a_{i,j}; \lambda_i(a_{i,j}), \gamma_i)$ is the proportion positive in age group j according to the model (equation (4)) (with $a_{i,j}$ taken as the mid-point of the the jth age group in study i), and $n_{i,j}$ and $k_{i,j}$ are the total number of individuals and the number that tested positive in age group j in study i.

We also fitted the model with a constant ROI and with an age-dependent ROI to the DAT seroprevalence and seroconversion data from Hasker et al [1] to confirm that the age-dependent conversion rate provides a better fit to this data. Given the form of the model, the number of seroconversions in age group j is Poisson distributed with rate parameter $\lambda(a_j)s(a_j)m_j$ (where m_j is the number of individuals in age group j), so the overall log-likelihood of the data is

$$L_H(b_0, b_1, \gamma) = \prod_{j=1}^{N} p_j^{k_j} (1 - p_j)^{n_j - k_j} \prod_{i=1}^{N} \lambda_j (1 - p_j) m_j e^{-\lambda_j (1 - p_j) m_j}. \tag{9}$$

All code was developed in MATLAB R2016b [2] and is freely available at `https://github.com/LloydChapman/VLageTrendsAnalysis`. The `mle` function in `MATLAB`'s Statistics and Machine Learning Toolbox was used to find the maximum likelihood estimates (MLEs) and, where appropriate, calculate their approximate 95% confidence intervals (CIs) using the Hessian of the log-likelihood surface at the MLE, approximating the surface as Normal (confidence intervals were not calculated where the likelihood surface was non-Normal).

1.3 Model comparison

The different models in Table 1 were compared in terms of their ability to fit the data using the Akaike information criterion (AIC), calculated from the overall likelihood L as:

$$\mathrm{AIC} = -2 \log L + 2N_p, \tag{10}$$

where N_p is the total number of parameters in the model. The model with the lowest AIC was selected as the best-fitting model.

2 Results

2.1 Hasker et al (2013) data

The results of fitting the age-independent and age-dependent ROI models to just the seroprevalence and seroconversion data from Hasker et al [1] are shown in Figure 1. The age-dependent model (bottom) has a much lower AIC (ΔAIC = 312.9) and is clearly a much better fit to the data than the age-independent model (top). Thus, it made sense to test whether the age-dependent ROI model was a better fit across all the datasets than the age-independent model.

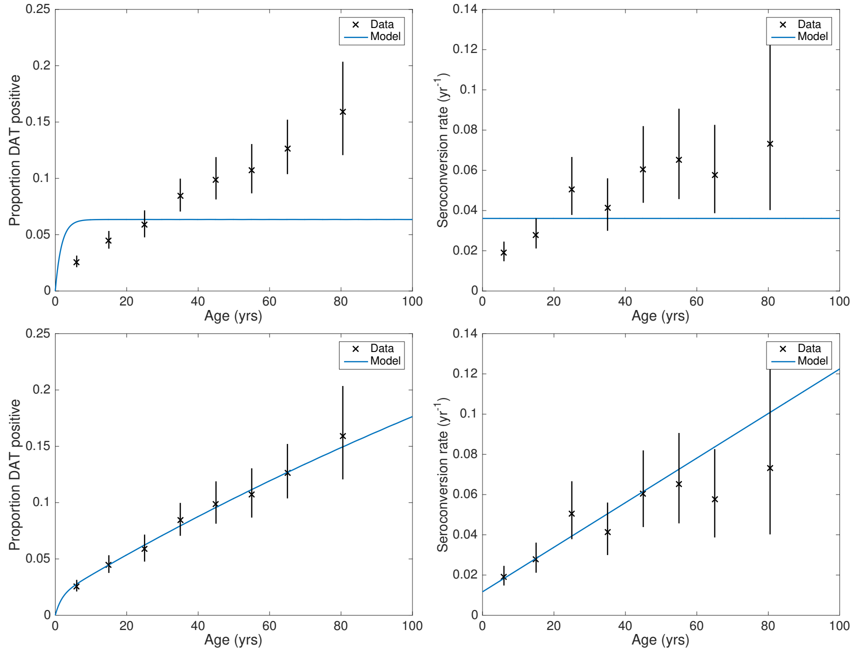

Fig 1. Fits of catalytic models with age-independent force of infection $\lambda(a) = b_0$ (top), and age-dependent force of infection $\lambda(a) = b_0 + b_1 a$ (bottom), to data on DAT prevalence (left) and seroconversion incidence (right) from Hasker et al [1]. Vertical lines show binomial and Poisson confidence intervals for the prevalence and seroconversion incidence in each age group.

2.2 Model selection

The overall AICs for the different models (Table 1) fitted to the data from all the infection prevalence studies in Table 3 in the main text are presented in Table 2. The best-fitting model is Model 6, the age-independent, study-specific conversion and reversion rate model. This model provides a far better fit to the data than any of the other age-independent or age-dependent models with other combinations of test- and/or study-specific conversion and reversion rates, except for Model 6a, the age-dependent model with study-specific rates, for which ΔAIC=6.5. The fact that the models with study-specific rates fit the data best suggests that the main source of variation in the rate estimates is the study that the data comes from, as opposed to the type of test used. This is likely to be due to genuine differences in the infection rate between different locations and time periods, e.g. with differences in clinical VL incidence, but may also reflect differences in test standardisation and protocols between studies. Although it is not obvious why the reversion rate should depend on the study, the study may be a proxy for other factors that affect the reversion rate, such as previous exposure and time since infection.

Table 2. Akaike information criterion (AIC) values for the different models fitted to the data and the number of fitted parameters in each model, N_p

Model	N_p	AIC	ΔAIC=AIC-AIC$_{\min}$
Age-independent, $\lambda_i(a) = b_{0,i}$ $(b_{1,i} = 0)$			
1	2	32905.1	2246.8
2	5	32535.4	1877.1
3	20	34220.5	3562.2
4	8	32313.0	1654.7
5	23	31425.4	767.1
6	38	30658.3	-
Age-dependent, $\lambda_i(a) = b_{0,i} + b_{1,i}a$			
1a	3	32867.3	2209.0
2a	9	32418.0	1759.7
3a	39	32807.9	2149.6
4a	12	32265.9	1607.6
5a	42	30806.3	148.0
6a	57	30664.8	6.5

$*$ ROI = Rate of infection

2.3 Parameter estimates

The ROI and reversion rate estimates and corresponding AIC value for each of the studies/study combinations for the different models are shown in Tables 3-4. Figure 3 shows the fits of the best-fitting model, Model 6 (the age-independent model with study-specific rates), for the individual studies. The fits are reasonable with the prevalence estimated from the model being within the confidence intervals of all the prevalence point estimates for the different age groups in the data for nearly all the studies. However, the model does not provide a good description of the trends in some studies, e.g. Bern et al, 2007 [3].

Although Model 6 is a better fit than Model 6a (its age-dependent equivalent) overall, the differences in the AIC values are small for most of the studies (ΔAIC ≤ 2), for 4 of the larger studies (Hasker et al [1] DAT and rK39, Singh et al [4] DAT, and Bern et al [5] LST) the AIC is actually lower for Model 6a, and Model 6a has a higher likelihood. Given this and the fact that the rate estimates from the age-dependent model agree more closely with those from longitudinal data (see Figure 2 and main text), we cannot discount the possibility that the ROI increases with age. However, there are several other potentially important factors, such as spatial variation in the ROI, that have not been taken into account.

Table 3. Conversion and reversion rate estimates for age-independent catalytic models

**Study**	**Location**	**Year**	**Test**	$b_{0,i}$	**95% CI**	$b_{1,i}$	**95% CI**	γ_i	**95% CI**	$-\log L$	AIC
Model 1											
All data	-	-	All	0.011	(0.011–0.012)	0	-	0.066	-(0.059–0.074)	16450.5	32905.1
Model 2											
DAT studies	-	-	DAT	0.016	(0.015–0.019)	0	-	0.12	(0.11–0.14)	9210.5	-
rK39 studies	-	-	rK39	0.015	(0.013–0.017)	0	-	0.12	(0.11–0.14)	5306.3	-
LST studies	-	-	LST	0.067	(0.058–0.077)	0	-	0.12	(0.11–0.14)	1464.9	-
PCR studies	-	-	PCR	0.0067	(0.0044–0.01)	0	-	0.12	(0.11–0.14)	281.0	-
Total										16262.7	32535.4
Model 3											
Hasker et al, 2013 [1]	Bihar, India	2009	DAT	0.035	∗	0	-	0.35	∗	2965.5	-
Koirala et al, 2004 [6]	Province No.1, Nepal	1996	DAT	0.0054	∗	0	-	0.35	∗	213.1	-
Ostyn et al, 2015 [7]	Province No.1, Nepal	2014	DAT	0.0054	∗	0	-	0.35	∗	174.1	-
Rijal et al, 2010 [8]	Province No.1 and No.2, Nepal	2006	DAT	0.029	∗	0	-	0.35	∗	1640.5	-
Schenkel et al, 2006 [9]	Province No.1, Nepal	2003	DAT	0.0025	∗	0	-	0.35	∗	141.6	-
Singh et al, [4]	Bihar, India	2006	DAT	0.074	∗	0	-	0.35	∗	3837.6	-
Topno et al, 2010 [10]	Bihar, India		DAT	0.0092	∗	0	-	0.35	∗	152.0	-
Bern et al, 2007 [3]	Mymensingh, Bangladesh	2002	rK39	0.02	∗	0	-	0.35	∗	949.7	-
Bern et al, 2007 [3]	Mymensingh, Bangladesh	2003	rK39	0.011	∗	0	-	0.35	∗	1015.0	-
Bern et al, 2007 [3]	Mymensingh, Bangladesh	2004	rK39	0.026	∗	0	-	0.35	∗	768.1	-
Hasker et al, 2013 [1]	Bihar, India	2009	rK39	0.038	∗	0	-	0.35	∗	2920.3	-
Topno et al, 2010 [10]	Bihar, India		rK39	0.0026	∗	0	-	0.35	∗	121.0	-
Bern et al, 2006 [5]	Mymensingh, Bangladesh	2002	LST	0.057	∗	0	-	0.35	∗	1192.9	-
Nandy et al, 1987 [11]	West Bengal, India		LST	0.0033	∗	0	-	0.35	∗	114.1	-
Patil et al, 2013 [12]	Jharkhand, India	2000	LST	0.0096	∗	0	-	0.35	∗	161.8	-
Schenkel et al, 2006 [9]	Province No.1, Nepal	2003	LST	0.011	∗	0	-	0.35	∗	179.6	-
Yangzom et al, 2012 [13]	, Bhutan	2011	LST	0.0016	∗	0	-	0.35	∗	234.1	-
Kaushal et al, 2017 [14]	West Bengal, India	2014	PCR	0.016	∗	0	-	0.35	∗	181.0	-
Topno et al, 2010 [10]	Bihar, India		PCR	0.0044	∗	0	-	0.35	∗	128.5	-
Total										17090.3	34220.5

Model 4											
DAT studies	-	-	DAT	0.01	(0.0093–0.011)	0	-	0.06	(0.051–0.07)	9171.4	18346.9
rK39 studies	-	-	rK39	0.011	(0.0093–0.012)	0	-	0.084	(0.068–0.1)	5298.1	10600.1
LST studies	-	-	LST	0.029	(0.024–0.037)	0	-	0.053	(0.038–0.074)	1438.7	2881.4
PCR studies	-	-	PCR	0.02	(0.0092–0.044)	0	-	0.11	(0.04–0.28)	240.3	484.7
Total										16148.5	32313.0
Model 5											
Hasker et al, 2013 [1]	Bihar, India	2009	DAT	0.023	∗	0	-	0.34	∗	2901.2	-
Koirala et al, 2004 [6]	Province No.1, Nepal	1996	DAT	0.0083	∗	0	-	0.34	∗	200.8	-
Ostyn et al, 2015 [7]	Province No.1, Nepal	2014	DAT	0.016	∗	0	-	0.34		142.4	-
Rijal et al, 2010 [8]	Province No.1 and No.2, Nepal	2006	DAT	0.029	∗	0	-	0.34	∗	1638.0	-
Schenkel et al, 2006 [9]	Province No.1, Nepal	2003	DAT	0.0096	∗	0	-	0.34	∗	110.1	-
Singh et al, [4]	Bihar, India	2006	DAT	0.085	∗	0	-	0.34	∗	3831.5	-
Topno et al, 2010 [10]	Bihar, India		DAT	0.046	∗	0	-	0.34	∗	122.7	-
Bern et al, 2007 [3]	Mymensingh, Bangladesh	2002	rK39	0.073	∗	0	-	0.31	∗	769.0	-
Bern et al, 2007 [3]	Mymensingh, Bangladesh	2003	rK39	0.056	∗	0	-	0.31	∗	771.9	-
Bern et al, 2007 [3]	Mymensingh, Bangladesh	2004	rK39	0.051	∗	0	-	0.31	∗	719.7	-
Hasker et al, 2013 [1]	Bihar, India	2009	rK39	0.02	∗	0	-	0.31	∗	2800.0	-
Topno et al, 2010 [10]	Bihar, India		rK39	0.026	∗	0	-	0.31	∗	87.7	-
Bern et al, 2006 [5]	Mymensingh, Bangladesh	2002	LST	0.08	∗	0	-	0.14	∗	963.3	-
Nandy et al, 1987 [11]	West Bengal, India		LST	0.044	∗	0	-	0.14	∗	59.7	-
Patil et al, 2013 [12]	Jharkhand, India	2000	LST	0.13	∗	0	-	0.14	∗	64.4	-
Schenkel et al, 2006 [9]	Province No.1, Nepal	2003	LST	0.025	∗	0	-	0.14	∗	143.4	-
Yangzom et al, 2012 [13]	, Bhutan	2011	LST	0.022	∗	0	-	0.14	∗	135.5	-
Kaushal et al, 2017 [14]	West Bengal, India	2014	PCR	0.99	(0.78–1.3)	0	-	3.5	(3.1–3.8)	130.7	-
Topno et al, 2010 [10]	Bihar, India		PCR	0.3	(0.2–0.43)	0	-	3.5	(3.1–3.8)	98.0	-
Total										15689.7	31425.4
Model 6											
Hasker et al, 2013 [1]	Bihar, India	2009	DAT	0.0037	(0.0032–0.0044)	0	-	0.021	(0.013–0.032)	2792.8	5589.5
Koirala et al, 2004 [6]	Province No.1, Nepal	1996	DAT	0.0059	(0.0021–0.017)	0	-	0.11	(0.032–0.35)	191.2	386.3
Ostyn et al, 2015 [7]	Province No.1, Nepal	2014	DAT	0.02	(0.0036–0.12)	0	-	0.18	(0.025–1.3)	131.6	267.2

Rijal et al, 2010 [8]	Province No.1 and No.2, Nepal	2006	DAT	0.008	(0.0062–0.01)	0	-	0.053	(0.037–0.077)	1603.7	3211.4
Schenkel et al, 2006 [9]	Province No.1, Nepal	2003	DAT	0.0073	(0.0023–0.023)	0	-	0.071	(0.017–0.3)	97.3	198.7
Singh et al, [4]	Bihar, India	2006	DAT	0.026	(0.022–0.03)	0	-	0.083	(0.067–0.1)	3775.0	7554.0
Topno et al, 2010 [10]	Bihar, India		DAT	0.019	(0.006–0.059)	0	-	0.13	(0.032–0.52)	122.3	248.5
Bern et al, 2007 [3]	Mymensingh, Bangladesh	2002	rK39	0.11	(0.072–0.18)	0	-	0.49	(0.31–0.76)	768.8	1541.5
Bern et al, 2007 [3]	Mymensingh, Bangladesh	2003	rK39	0.059	(0.035–0.1)	0	-	0.32	(0.19–0.56)	771.9	1547.8
Bern et al, 2007 [3]	Mymensingh, Bangladesh	2004	rK39	0.032	(0.018–0.055)	0	-	0.19	(0.1–0.36)	718.9	1441.8
Hasker et al, 2013 [1]	Bihar, India	2009	rK39	0.0034	(0.0029–0.0039)	0	-	0.017	(0.011–0.028)	2682.9	5369.9
Topno et al, 2010 [10]	Bihar, India		rK39	0.026	(0.0059–0.12)	0	-	0.35	(0.073–1.6)	87.6	179.2
Bern et al, 2006 [5]	Mymensingh, Bangladesh	2002	LST	0.028	(0.023–0.035)	0	-	0.023	(0.014–0.037)	928.3	1860.6
Nandy et al, 1987 [11]	West Bengal, India		LST	0.011	(0.0025–0.02)	0	-	0.0027	(0–0.033)	56.3	116.6
Patil et al, 2013 [12]	Jharkhand, India	2000	LST	0.056	(0.032–0.099)	0	-	0.035	(0.013–0.095)	60.7	125.4
Schenkel et al, 2006 [9]	Province No.1, Nepal	2003	LST	0.53	(0.39–0.71)	0	-	3.5	(3–4)	142.1	288.1
Yangzom et al, 2012 [13]	Bhutan	2011	LST	0.0086	(0.0039–0.019)	0	-	0.051	(0.016–0.16)	134.2	272.4
Kaushal et al, 2017 [14]	West Bengal, India	2014	PCR	0.021	(0.01–0.042)	0	-	0.053	(0.019–0.15)	127.7	259.4
Topno et al, 2010 [10]	Bihar, India		PCR	0.71	(0.51–0.99)	0	-	8.3	(7.9–8.7)	98.0	200.0
Total										15291.2	30658.3

∗ Confidence intervals not calculated as Normal approximation to the Hessian of the log-likelihood surface at the maximum likelihood estimate is inappropriate due to non-normality of the likelihood surface.

Table 4. Conversion and reversion rate estimates for age-dependent catalytic models

**Study**	**Location**	**Year**	**Test**	$b_{0,i}$	**95% CI**	$b_{1,i}$	**95% CI**	γ_i	**95% CI**	$-\log L$	AIC
Model 1a											
All data	-	-	All	0.014	(0.013–0.016)	0.00019	(0.00012–0.00029)	0.13	(0.11–0.17)	16430.6	32867.3
Model 2a											
DAT studies	-	-	DAT	0.024	$*$	0.00087	$*$	0.39	$*$	9152.9	18323.8
rK39 studies	-	-	rK39	0.025	$*$	0.0006	$*$	0.39	$*$	5314.4	10646.8
LST studies	-	-	LST	0.0026	$*$	0.0065	$*$	0.39	$*$	1489.3	2996.7
PCR studies	-	-	PCR	0.03	$*$	0.00072	$*$	0.39	$*$	243.4	504.7
Total										16200.0	32472.0
Model 3a											
Hasker et al, 2013 [1]	Bihar, India	2009	DAT	0.0099	$*$	0.0009	$*$	0.49	$*$	2789.5	5657.0
Koirala et al, 2004 [6]	Province No.1, Nepal	1996	DAT	0.012	$*$	0.00091	$*$	0.49	$*$	202.7	483.4
Ostyn et al, 2015 [7]	Province No.1, Nepal	2014	DAT	0.0086	$*$	0.00098	$*$	0.49	$*$	140.0	358.1
Rijal et al, 2010 [8]	Province No.1 and No.2, Nepal	2006	DAT	0.0095	$*$	0.00099	$*$	0.49	$*$	1635.8	3349.7
Schenkel et al, 2006 [9]	Province No.1, Nepal	2003	DAT	0.0098	$*$	0.00093	$*$	0.49	$*$	99.1	276.1
Singh et al, [4]	Bihar, India	2006	DAT	0.01	$*$	0.0042	$*$	0.49	$*$	3934.5	7947.0
Topno et al, 2010 [10]	Bihar, India		DAT	0.01	$*$	0.00092	$*$	0.49	$*$	132.0	342.0
Bern et al, 2007 [3]	Mymensingh, Bangladesh	2002	rK39	0.0094	$*$	0.00098	$*$	0.49	$*$	960.6	1999.1
Bern et al, 2007 [3]	Mymensingh, Bangladesh	2003	rK39	0.0095	$*$	0.0012	$*$	0.49	$*$	895.2	1868.5
Bern et al, 2007 [3]	Mymensingh, Bangladesh	2004	rK39	0.0095	$*$	0.00096	$*$	0.49	$*$	816.9	1711.9
Hasker et al, 2013 [1]	Bihar, India	2009	rK39	0.0094	$*$	0.00088	$*$	0.49	$*$	2684.3	5446.6
Topno et al, 2010 [10]	Bihar, India		rK39	0.0094	$*$	0.00097	$*$	0.49	$*$	92.7	263.4
Bern et al, 2006 [5]	Mymensingh, Bangladesh	2002	LST	0.0097	$*$	0.0025	$*$	0.49	$*$	1221.4	2520.9
Nandy et al, 1987 [11]	West Bengal, India		LST	0.0088	$*$	0.00094	$*$	0.49	$*$	71.1	220.2
Patil et al, 2013 [12]	Jharkhand, India	2000	LST	0.0098	$*$	0.00095	$*$	0.49	$*$	126.7	331.4
Schenkel et al, 2006 [9]	Province No.1, Nepal	2003	LST	0.009	$*$	0.00093	$*$	0.49	$*$	156.6	391.2

Yangzom et al, 2012 [13]	, Bhutan	2011	LST	0.0093	∗	0.0009	∗	0.49	∗	138.8	355.6
Kaushal et al, 2017 [14]	West Bengal, India	2014	PCR	0.0097	∗	0.00093	∗	0.49	∗	158.7	395.5
Topno et al, 2010 [10]	Bihar, India		PCR	0.0072	∗	0.00094	∗	0.49	∗	108.2	294.5
Total										16364.9	34211.9
Model 4a											
DAT studies	-	-	DAT	0.015	(0.013–0.019)	0.00033	(0.0002–0.00053)	0.18	(0.13–0.25)	9146.2	18298.4
rK39 studies	-	-	rK39	0.011	(0.0093–0.014)	2.9e-05	(2.4e-06–0.00036)	0.097	(0.065–0.14)	5297.7	10601.4
LST studies	-	-	LST	0.036	(0.026–0.05)	0.00052	(0.00013–0.0021)	0.11	(0.052–0.22)	1436.7	2879.5
PCR studies	-	-	PCR	0.02	(0.0092–0.044)	0	-	0.11	(0.04–0.28)	240.3	486.7
Total										16121.0	32265.9
Model 5a											
Hasker et al, 2013 [1]	Bihar, India	2009	DAT	0.0087	∗	0.00094	∗	0.44	∗	2788.4	5606.7
Koirala et al, 2004 [6]	Province No.1, Nepal	1996	DAT	0.0083	∗	0.00046	∗	0.44	∗	192.0	414.0
Ostyn et al, 2015 [7]	Province No.1, Nepal	2014	DAT	0.0097	∗	0.0012	∗	0.44	∗	138.3	306.6
Rijal et al, 2010 [8]	Province No.1 and No.2, Nepal	2006	DAT	0.0088	∗	0.0013	∗	0.44	∗	1621.8	3273.5
Schenkel et al, 2006 [9]	Province No.1, Nepal	2003	DAT	0.0076	∗	0.00096	∗	0.44	∗	99.2	228.4
Singh et al, [4]	Bihar, India	2006	DAT	0.064	∗	0.0015	∗	0.44	∗	3780.1	7590.2
Topno et al, 2010 [10]	Bihar, India		DAT	0.0066	∗	0.0012	∗	0.44	∗	131.1	292.3
Bern et al, 2007 [3]	Mymensingh, Bangladesh	2002	rK39	0.086	∗	0.00012	∗	0.42	∗	770.2	1562.4
Bern et al, 2007 [3]	Mymensingh, Bangladesh	2003	rK39	0.069	∗	0.00019	∗	0.42	∗	773.8	1569.7
Bern et al, 2007 [3]	Mymensingh, Bangladesh	2004	rK39	0.062	∗	4.8e-05	∗	0.42	∗	720.6	1463.3
Hasker et al, 2013 [1]	Bihar, India	2009	rK39	0.0074	∗	0.00079	∗	0.42	∗	2683.5	5388.9
Topno et al, 2010 [10]	Bihar, India		rK39	0.011	∗	0.0012	∗	0.42	∗	93.4	208.7
Bern et al, 2006 [5]	Mymensingh, Bangladesh	2002	LST	0.00067	∗	0.0093	∗	0.32	∗	942.7	1907.4
Nandy et al, 1987 [11]	West Bengal, India		LST	0.02	∗	0.0038	∗	0.32	∗	55.9	133.8
Patil et al, 2013 [12]	Jharkhand, India	2000	LST	0.029	∗	0.014	∗	0.32	∗	62.7	147.4

Schenkel et al, 2006 [9]	Province No.1, Nepal	2003	LST	0.021	∗	0.00064	∗	0.32	∗	145.8	313.6
Yangzom et al, 2012 [13]	, Bhutan	2011	LST	0.017	∗	0.00078	∗	0.32	∗	134.4	290.7
Kaushal et al, 2017 [14]	West Bengal, India	2014	PCR	0.31	(0.1–0.95)	0.009	(0.002–0.04)	2.1	(1.2–3.6)	129.4	268.7
Topno et al, 2010 [10]	Bihar, India		PCR	0.18	(0.083–0.38)	0	-	2.1	(1.2–3.6)	98.0	206.0
Total										15361.2	31172.3
Model 6a											
Hasker et al, 2013 [1]	Bihar, India	2009	DAT	0.008	(0.0049–0.013)	0.00075	(0.00034–0.0016)	0.37	(0.18–0.77)	2788.1	5582.1
Koirala et al, 2004 [6]	Province No.1, Nepal	1996	DAT	0.017	(0.00036–0.78)	0.00041	(3.2e-06–0.052)	0.67	(0.0075–59)	190.8	387.5
Ostyn et al, 2015 [7]	Province No.1, Nepal	2014	DAT	0.02	∗	0	-	0.18	∗	131.6	269.2
Rijal et al, 2010 [8]	Province No.1 and No.2, Nepal	2006	DAT	0.0096	(0.0062–0.015)	9.5e-05	(1.3e-05–0.0007)	0.097	(0.038–0.25)	1603.0	3212.0
Schenkel et al, 2006 [9]	Province No.1, Nepal	2003	DAT	0.0073	(0.0023–0.023)	0	-	0.071	(0.017–0.3)	97.3	200.7
Singh et al, [4]	Bihar, India	2006	DAT	0.032	(0.025–0.039)	0.00031	(0.00014–0.00069)	0.15	(0.1–0.21)	3769.2	7544.5
Topno et al, 2010 [10]	Bihar, India		DAT	0.019	(0.006–0.059)	0	-	0.13	(0.032–0.52)	122.3	250.5
Bern et al, 2007 [3]	Mymensingh, Bangladesh	2002	rK39	0.13	∗	0	-	0.56	∗	768.8	1543.5
Bern et al, 2007 [3]	Mymensingh, Bangladesh	2003	rK39	0.059	∗	0	-	0.32	∗	771.9	1549.8
Bern et al, 2007 [3]	Mymensingh, Bangladesh	2004	rK39	0.032	∗	0	-	0.19	∗	718.9	1443.8
Hasker et al, 2013 [1]	Bihar, India	2009	rK39	0.0044	(0.0027–0.0071)	0.00025	(3.9e-05–0.0016)	0.13	(0.027–0.67)	2681.7	5369.4
Topno et al, 2010 [10]	Bihar, India		rK39	0.026	(0.0059–0.12)	0	-	0.35	(0.073–1.6)	87.6	181.2
Bern et al, 2006 [5]	Mymensingh, Bangladesh	2002	LST	0.038	(0.025–0.056)	0.002	(0.00048–0.0085)	0.13	(0.041–0.39)	926.4	1858.7
Nandy et al, 1987 [11]	West Bengal, India		LST	0.094	(0.021–0.43)	0.013	(0.0064–0.026)	1.4	(1.1–1.9)	55.4	116.8
Patil et al, 2013 [12]	Jharkhand, India	2000	LST	0.056	∗	0	-	0.035	∗	60.7	127.4
Schenkel et al, 2006 [9]	Province No.1, Nepal	2003	LST	0.27	(0.2–0.38)	0	-	1.8	(1.5–2.2)	142.1	290.1

Yangzom et al, 2012 [13]	, Bhutan	2011	LST	0.011	(0.0021–0.056)	0.00012	(2.5e-07–0.056)	0.1	(0.0045–2.3)	134.1	274.2
Kaushal et al, 2017 [14]	West Bengal, India	2014	PCR	0.021	∗	0	-	0.053	∗	127.7	261.4
Topno et al, 2010 [10]	Bihar, India		PCR	0.33	(0.22–0.48)	0	-	3.8	(3.2–4.6)	98.0	202.0
Total										15275.4	30664.8

∗ Confidence intervals not calculated as Normal approximation to the Hessian of the log-likelihood surface at the maximum likelihood estimate is inappropriate due to non-normality of the likelihood surface.

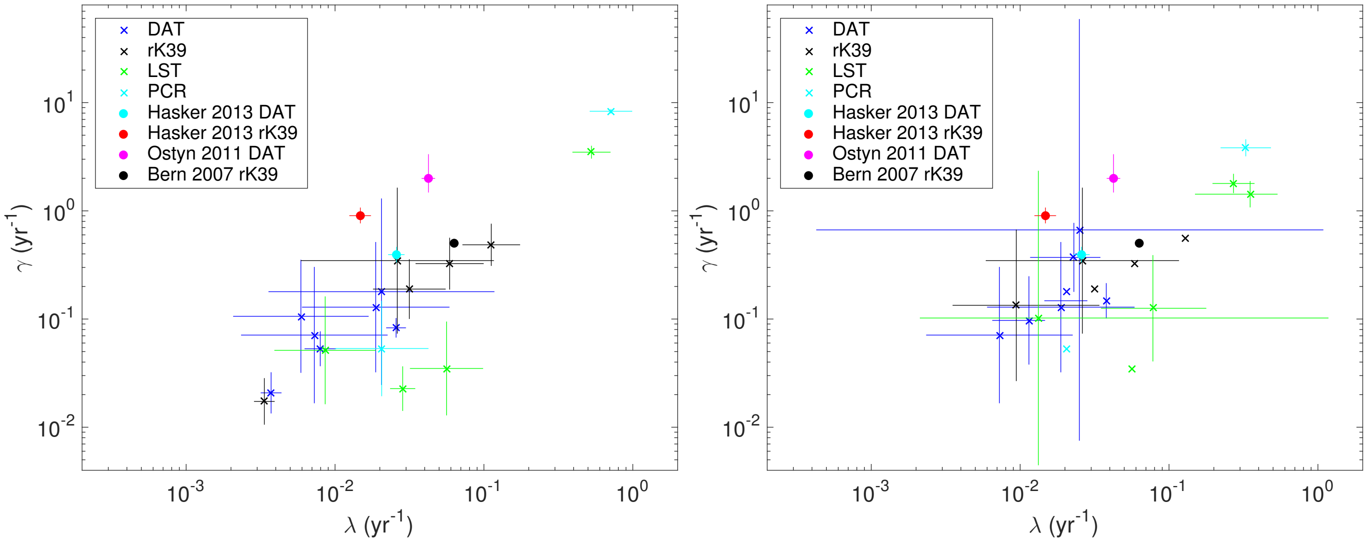

Fig 2. Estimated conversion and reversion rates from (a) the age-independent model with study-specific rates (Model 6), and (b) the age-dependent model with study-specific rates (Model 6a) at age 20 yrs.

DAT

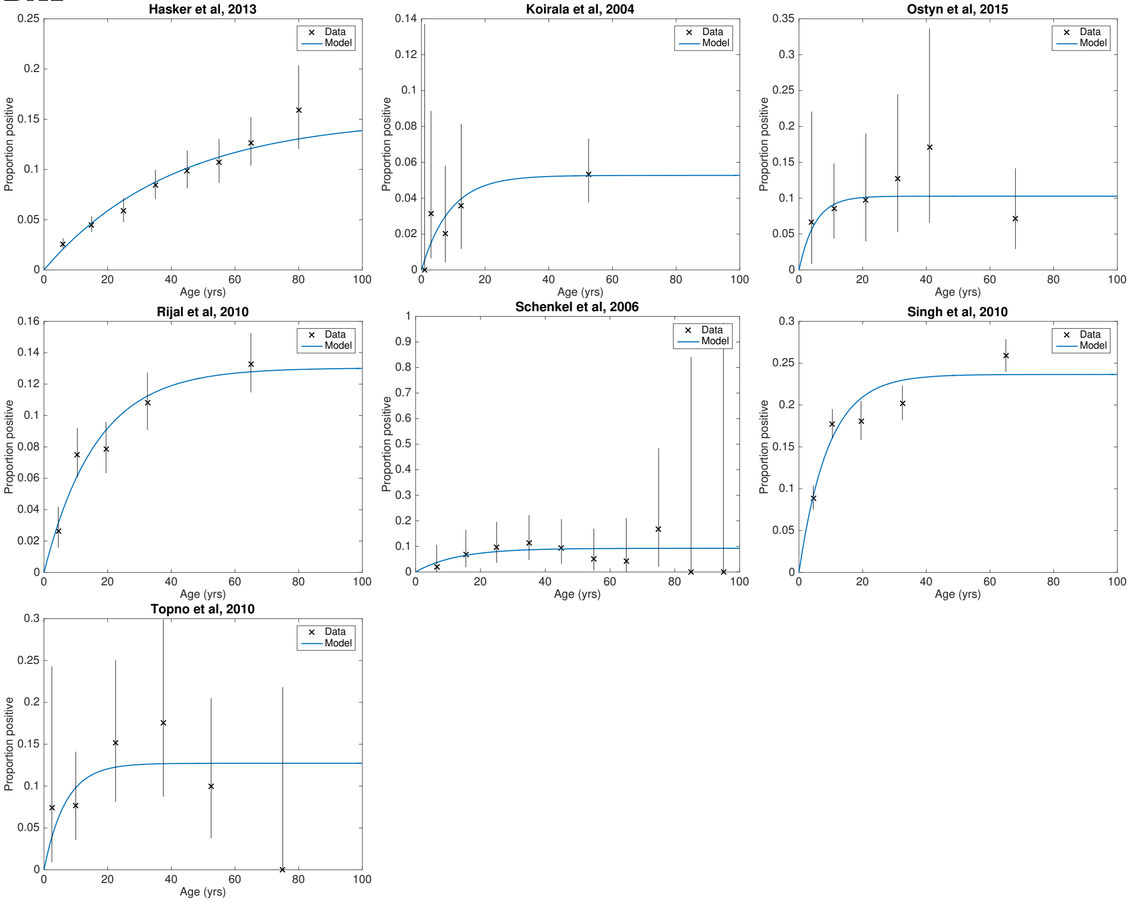
Hasker et al, 2013
Koirala et al, 2004
Ostyn et al, 2015
Rijal et al, 2010
Schenkel et al, 2006
Singh et al, 2010
Topno et al, 2010
Data
Model
Proportion positive
Age (yrs)

rK39

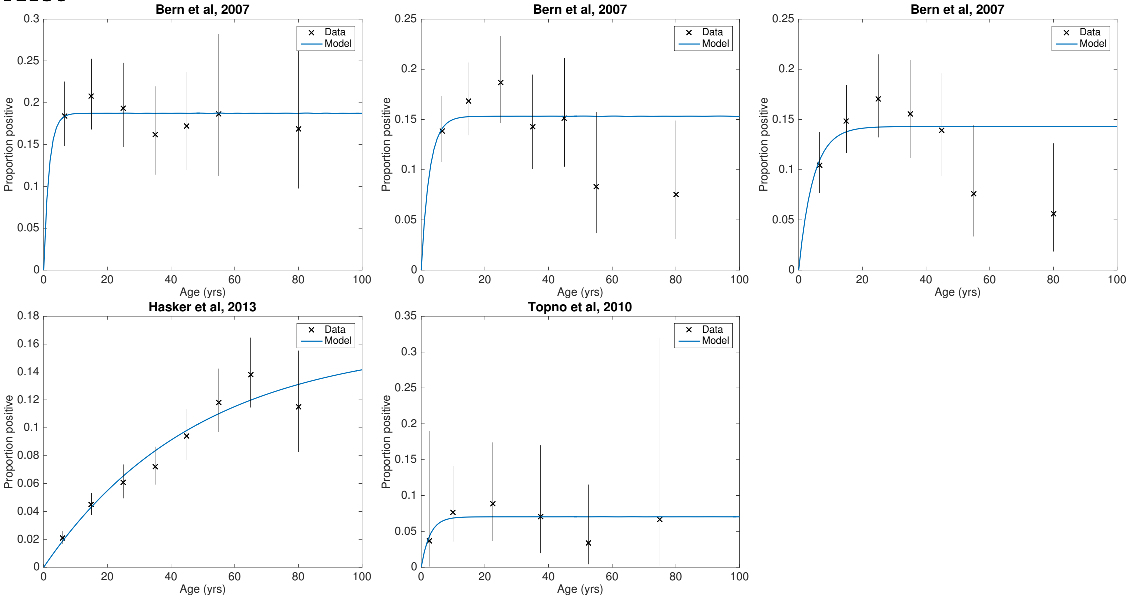
Bern et al, 2007
Bern et al, 2007
Bern et al, 2007
Hasker et al, 2013
Topno et al, 2010
Data
Model
Proportion positive
Age (yrs)

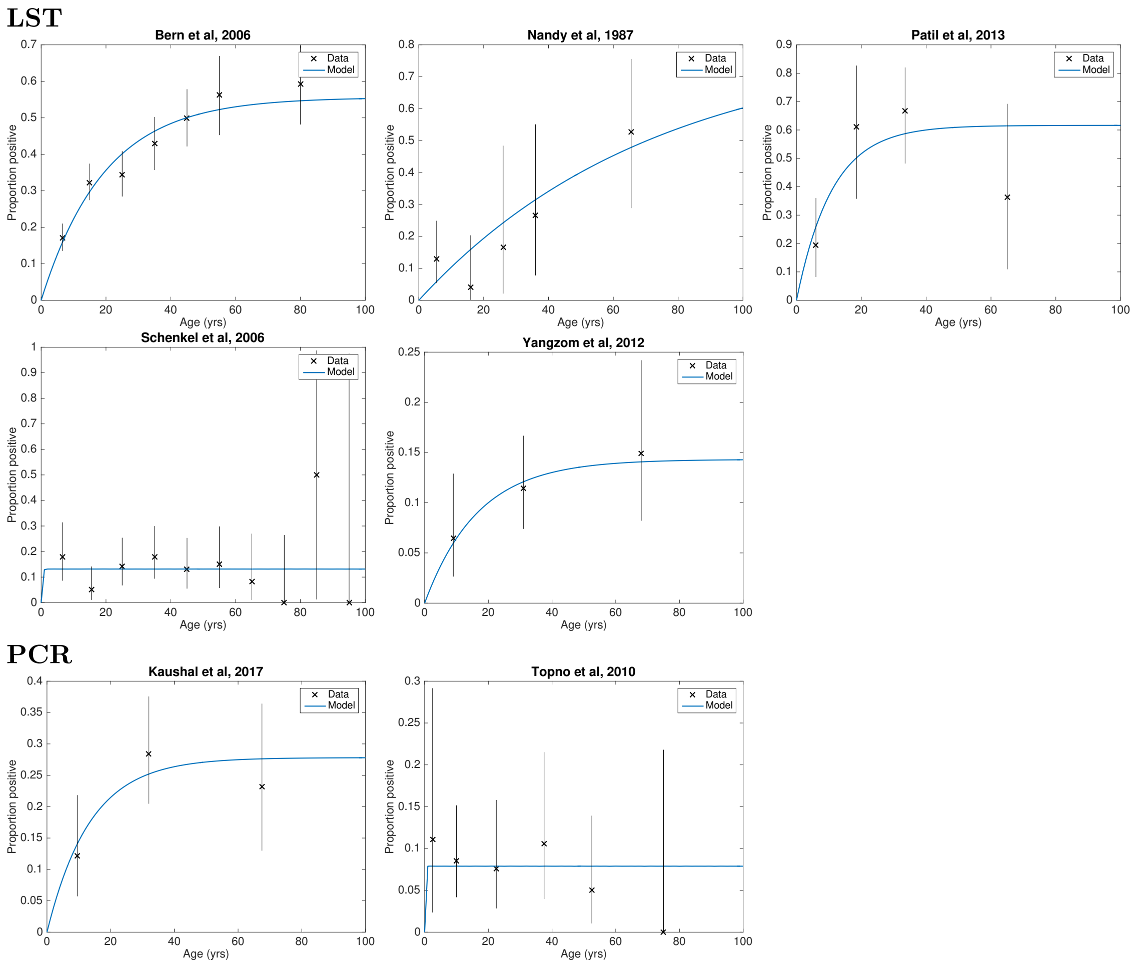

Fig 3. Fits of age-independent model (Model 6) to individual studies.

References

1. Hasker E, Kansal S, Malaviya P, Gidwani K, Picado A, Singh RP, et al. Latent infection with Leishmania donovani in highly endemic villages in Bihar, India. PLoS Neglected Tropical Diseases. 2013;7(2):e2053. doi:10.1371/journal.pntd.0002053.
2. MATLAB, Statistics and Machine Learning Toolbox, and Optimization Toolbox R2016b (9.1.0). The MathWorks, Inc. Natick, Massachusetts, United States; 2015.
3. Bern C, Haque R, Chowdhury R, Ali M, Kurkjian KM, Vaz L, et al. The epidemiology of visceral leishmaniasis and asymptomatic leishmanial infection in a highly endemic Bangladeshi village. American Journal of Tropical Medicine and Hygiene. 2007;76(5):909–914.
4. Singh SP, Picado A, Boelaert M, Gidwani K, Andersen EW, Ostyn B, et al. The epidemiology of Leishmania donovani infection in high transmission foci in India. Tropical Medicine and International Health. 2010;15(Suppl. 2):12–20. doi:10.1111/j.1365-3156.2010.02519.x.
5. Bern C, Amann J, Haque R, Chowdhury R, Ali M, Kurkjian KM, et al. Loss of leishmanin skin test antigen sensitivity and potency in a longitudinal study of visceral leishmaniasis in Bangladesh. American Journal of Tropical Medicine and Hygiene. 2006;75(4):744–8.
6. Koirala S, Karki P, Das ML, Parija SC, Karki BMS. Epidemiological study of kala-azar by direct agglutination test in two rural communities of eastern Nepal. Tropical Medicine and International Health. 2004;9(4):533–537. doi:10.1111/j.1365-3156.2004.01212.x.
7. Ostyn B, Uranw S, Bhattarai NR, Das ML, Rai K, Tersago K, et al. Transmission of Leishmania donovani in the Hills of Eastern Nepal, an Outbreak Investigation in Okhaldhunga and Bhojpur Districts. PLoS Neglected Tropical Diseases. 2015;9(8):e0003966. doi:10.1371/journal.pntd.0003966.
8. Rijal S, Uranw S, Chappuis F, Picado A, Khanal B, Paudel IS, et al. Epidemiology of Leishmania donovani infection in high-transmission foci in Nepal. Tropical Medicine and International Health. 2010;15(Suppl. 2):21–28. doi:10.1111/j.1365-3156.2010.02518.x.
9. Schenkel K, Rijal S, Koirala S, Koirala S, Vanlerberghe V, Van Der Stuyft P, et al. Visceral leishmaniasis in southeastern Nepal: A cross-sectional survey on Leishmania donovani infection and its risk factors. Tropical Medicine and International Health. 2006;11(12):1792–1799. doi:10.1111/j.1365-3156.2006.01735.x.
10. Topno RK, Das VNR, Ranjan A, Pandey K, Singh D, Kumar N, et al. Asymptomatic infection with visceral leishmaniasis in a disease-endemic area in Bihar, India. American Journal of Tropical Medicine and Hygiene. 2010;83(3):502–506. doi:10.4269/ajtmh.2010.09-0345.
11. Nandy A, Neogy AB, Chowdhury AB. Leishmanin test survey in an endemic village of Indian kala-azar near Calcutta. Annals of Tropical Medicine & Parasitology. 1987;81(6):693–699.
12. Patil RR, Muliyil JP, Nandy A, Addy M, Maji A, Chatterjee P. Immuno-epidemiology of leishmanial infection among tribal population in kala-azar endemic areas: A community based study. 2013;6(1):2–7. doi:10.4103/1755-6783.115193.
13. Yangzom T, Cruz I, Bern C, Argaw D, den Boer M, Vélez ID, et al. Endemic transmission of visceral leishmaniasis in Bhutan. American Journal of Tropical Medicine and Hygiene. 2012;87(6):1028–37. doi:10.4269/ajtmh.2012.12-0211.
14. Kaushal H, Bhattacharya SK, Verma S, Salotra P. Serological and Molecular Analysis of Leishmania Infection in Healthy Individuals from Two Districts of West Bengal, India, Endemic for Visceral Leishmaniasis. American Journal of Tropical Medicine and Hygiene. 2017;96(6):1448–1455. doi:10.4269/ajtmh.16-0592.
