# Supplementary material for: Age trends in asymptomatic and symptomatic Leishmania donovani infection in the Indian subcontinent: A review and analysis of data from diagnostic and epidemiological studies
Source: PLoS Negl Trop Dis. 2018 Dec 6;12(12):e0006803. doi: 10.1371/journal.pntd.0006803 (PMC6283524; doi:10.1371/journal.pntd.0006803)
Supplement: S3 Text — (DOCX) [file pntd.0006803.s004.docx]

Table 1. Potential risk of bias in included cohort studies

| **Potential risk of bias*** **in** | | | | | | | | |
| --- | --- | --- | --- | --- | --- | --- | --- | --- |
| **Study** | **Selection of exposed cohort** | **Selection of non-exposed cohort** | **Exposure ascertainment** | **Outcome not present at start of study** | **Comparability of exposed and non-exposed cohort** | **Outcome ascertainment** | **Follow up not long enough** | **Loss to follow up** |
| Bern et al, 2005 [1] | No | No | No | No | No | No | Yes | No |
| Bern et al, 2007 [2] | No | No | No | No | No | No | No | No |
| Ferdousi et al, 2010 [3] | No | No | No | No | No | No | No | Yes |
| Hasker et al, 2012 [4] | No | No | No | No | No | No | No | Yes |
| Hasker et al, 2013 [5] | No | No | No | No | Yes | No | No | Yes |
| Singh et al, 2010 [6] | No | No | No | No | Yes | No | Yes | Yes |

* Assessed using Newcastle-Ottawa Scale [7].

Table 2. Potential risk of bias in included case-control studies

| **Potential risk of bias*** **in** | | | | | | | | |
| --- | --- | --- | --- | --- | --- | --- | --- | --- |
| **Study** | **Case selection** | **Case representativeness** | **Control selection** | **Control representativeness** | **Comparability of case and control** | **Exposure ascertainment** | **Exposure ascertainment different for control** | **Non-response rate** |
| Ostyn et al, 2015 [8] | No | No | No | No | No | No | No | Yes |

* Assessed using Newcastle-Ottawa Scale [7].

Table 3. Potential risk of bias in included experimental studies

| **Potential risk of bias*** **in** | | | | | | | |
| --- | --- | --- | --- | --- | --- | --- | --- |
| **Study** | **Allocation sequence generation** | **Allocation concealment** | **Blinding of participants** | **Blinding of investigator** | **Blinding of outcome assessor** | **Completeness of outcome data** | **Outcome reporting** |
| Bhattarai et al, 2009 [9] | Low | Unclear | Unclear | Unclear | Low | Low | Low |
| Picado et al, 2014 [10] | Low | Unclear | Unclear | Unclear | Low | Low | Low |
| Rijal et al, 2010 [11] | Low | Unclear | Unclear | Unclear | Low | Low | Low |

* Assessed using Cochrance risk of bias assessment tool [12].

Table 4. Potential risk of bias in included cross-sectional studies

| **Potential risk of bias*** **in** | | | | | | | |
| --- | --- | --- | --- | --- | --- | --- | --- |
| **Study** | **Sample representativeness** | **Sample size justified** | **Comparability of non-respondents** | **Exposure ascertainment** | **Comparability of different outcome groups** | **Outcome assessment** | **Statistical appropriateness** |
| Bern et al, 2006 [13] | No | Yes | Yes | No | No | No | No |
| Kaushal et al, 2017 [14] | No | Yes | Yes | No | Yes | No | No |
| Koirala et al, 2004 [15] | Yes | Yes | Yes | No | Yes | No | No |
| Nandy et al, 1987 [16] | No | Yes | Yes | No | Yes | No | Yes |
| Patil et al, 2013 [17] | No | Yes | Yes | No | Yes | No | Yes |
| Schenkel et al, 2006 [18] | No | No | Yes | No | No | No | No |
| Singh et al, 2010 [19] | No | Yes | Yes | No | No | No | No |
| Topno et al, 2010 [20] | Yes | Yes | Yes | No | Yes | No | Yes |
| Yangzom et al, 2012 [21] | Yes | Yes | Yes | No | No | No | No |

* Assessed using adapted Newton-Ottawa Scale [22].

**References**

1. Bern C, Hightower AW, Chowdhury R, Ali M, Amann J, Wagatsuma Y, et al. Risk factors for kala-azar in Bangladesh. Emerg Infect Dis. 2005;11(5):655–62.

2. Bern C, Haque R, Chowdhury R, Ali M, Kurkjian KM, Vaz L, et al. The epidemiology of visceral leishmaniasis and asymptomatic leishmanial infection in a highly endemic Bangladeshi village. Am J Trop Med Hyg. 2007;76(5):909–14.

3. Ferdousi F, Alam MS, Hossain MS, Ma E, Itoh M, Mondal D, et al. Visceral Leishmaniasis Eradication is a Reality: Data from a Community-based Active Surveillance in Bangladesh. Trop Med Health. 2012;40(4):133–9.

4. Hasker E, Singh SP, Malaviya P, Picado A, Gidwani K, Singh RP, et al. Visceral Leishmaniasis, Rural Bihar, India. Emerg Infect Dis. 2012;18(10):1662–4. Available from: http://wwwnc.cdc.gov/eid/article/18/10/11-1083_article.htm

5. Hasker E, Kansal S, Malaviya P, Gidwani K, Picado A, Singh RP, et al. Latent infection with Leishmania donovani in highly endemic villages in Bihar, India. PLoS Negl Trop Dis. 2013;7(2):e2053.

6. Singh VP, Ranjan A, Topno RK, Verma RB, Siddique NA, Ravidas VN, et al. Short Report: Estimation of Under-Reporting of Visceral Leishmaniasis Cases in Bihar, India. Am J Trop Med Hyg. 2010;82(1):9–11. Available from: http://www.ajtmh.org/cgi/doi/10.4269/ajtmh.2010.09-0235

7. Wells GA, Shea B, O’Connell D, Peterson J, Welch V. Newcastle-Ottawa Scale (NOS) for assessing the quality of nonrandomised studies in meta-analyses. Available from: http://www.ohri.ca/programs/clinical_epidemiology/nosgen.pdf

8. Ostyn B, Uranw S, Bhattarai NR, Das ML, Rai K, Tersago K, et al. Transmission of Leishmania donovani in the Hills of Eastern Nepal, an Outbreak Investigation in Okhaldhunga and Bhojpur Districts. PLoS Negl Trop Dis. 2015;9(8):e0003966. Available from: http://dx.plos.org/10.1371/journal.pntd.0003966

9. Bhattarai NR, Auwera G Van Der, Khanal B, Doncker S De, Rijal S, Boelaert M, et al. PCR and direct agglutination as Leishmania infection markers among healthy Nepalese subjects living in areas endemic for Kala-Azar. Trop Med Int Heal. 2009;14(4):404–11.

10. Picado A, Ostyn B, Singh SP, Uranw S, Hasker E, Rijal S, et al. Risk factors for visceral leishmaniasis and asymptomatic Leishmania donovani infection in India and Nepal. PLoS One. 2014;9(1):1–8.

11. Rijal S, Uranw S, Chappuis F, Picado A, Khanal B, Paudel IS, et al. Epidemiology of Leishmania donovani infection in high-transmission foci in Nepal. Trop Med Int Heal. 2010;15(Suppl. 2):21–8.

12. Higgins J, Altman D, Sterne J. Chapter 8: Assessing risk of bias in included studies. In: Higgins J, Green S, editors. Cochrane Handbook for Systematic Reviews of Interventions Version 510. The Cochrane Collaboration, 2011; 2011. Available from: http://handbook-5-1.cochrane.org/

13. Bern C, Amann J, Haque R, Chowdhury R, Ali M, Kurkjian KM, et al. Loss of leishmanin skin test antigen sensitivity and potency in a longitudinal study of visceral leishmaniasis in Bangladesh. Am J Trop Med Hyg. 2006;75(4):744–8.

14. Kaushal H, Bhattacharya SK, Verma S, Salotra P. Serological and Molecular Analysis of Leishmania Infection in Healthy Individuals from Two Districts of West Bengal, India, Endemic for Visceral Leishmaniasis. Am J Trop Med Hyg. 2017;96(6):1448–55.

15. Koirala S, Karki P, Das ML, Parija SC, Karki BMS. Epidemiological study of kala-azar by direct agglutination test in two rural communities of eastern Nepal. Trop Med Int Heal. 2004;9(4):533–7.

16. Nandy A, Neogy AB, Chowdhury AB. Leishmanin test survey in an endemic village of Indian kala-azar near Calcutta. Ann Trop Med Parasitol. 1987;81(6):693–9.

17. Patil RR, Muliyil JP, Nandy A, Addy M, Maji A, Chatterjee P. Immuno-epidemiology of leishmanial infection among tribal population in kala-azar endemic areas: A community based study. Ann Trop Med Public Heal. 2013;6(1):50. Available from: http://www.atmph.org/text.asp?2013/6/1/50/115193

18. Schenkel K, Rijal S, Koirala SS, Koirala SS, Vanlerberghe V, Van der Stuyft P, et al. Visceral leishmaniasis in southeastern Nepal: A cross-sectional survey on Leishmania donovani infection and its risk factors. Trop Med Int Heal. 2006;11(12):1792–9.

19. Singh SP, Picado A, Boelaert M, Gidwani K, Andersen EW, Ostyn B, et al. The epidemiology of Leishmania donovani infection in high transmission foci in India. Trop Med Int Heal. 2010;15(Suppl. 2):12–20.

20. Topno RK, Das VNR, Ranjan A, Pandey K, Singh D, Kumar N, et al. Asymptomatic infection with visceral leishmaniasis in a disease-endemic area in Bihar, India. Am J Trop Med Hyg. 2010;83(3):502–6.

21. Yangzom T, Cruz I, Bern C, Argaw D, den Boer M, Vélez ID, et al. Endemic transmission of visceral leishmaniasis in Bhutan. Am J Trop Med Hyg. 2012;87(6):1028–37.

22. Herzog R, Álvarez-Pasquin MJ, Díaz C, Del Barrio JL, Estrada JM, Gil Á. Are healthcare workers’ intentions to vaccinate related to their knowledge, beliefs and attitudes? a systematic review. BMC Public Health. 2013;13(1):154. Available from: https://static-content.springer.com/esm/art%3A10.1186%2F1471-2458-13-154/MediaObjects/12889_2012_5111_MOESM3_ESM.doc
