# Supplementary material for: Age trends in asymptomatic and symptomatic Leishmania donovani infection in the Indian subcontinent: A review and analysis of data from diagnostic and epidemiological studies
Source: PLoS Negl Trop Dis. 2018 Dec 6;12(12):e0006803. doi: 10.1371/journal.pntd.0006803 (PMC6283524; doi:10.1371/journal.pntd.0006803)
Supplement: S4 Text — (DOCX) [file pntd.0006803.s005.docx]

**Descriptions of the diagnostic tests used in the identified studies**

**Direction agglutination test (DAT)**

The direct agglutination test (DAT) is a semi-quantitative test for antibodies against *L.donovani*. Increasing dilutions of the individual’s serum or blood eluted from filter paper are placed into micro-titre plates with V-shaped wells containing a freeze-dried suspension of killed and stained *L. donovani* promastigotes. If antibodies against *L. donovani* are present, a pale blue film forms over the well, while if they are absent, the DAT antigen accumulates at the bottom of the plate to form a dark blue spot [1]. The individual is deemed seropositive if the pale film still forms at or above a certain serum dilution (typically 1:1600), referred to as the cut-off titre.

**rK39 enzyme-linked immunosorbent assay (ELISA)**

rK39 (recombinant K39) is the cloned antigen of a 39-amino-acid repeat of a kinesin-like gene found in *Leishmania infantum* (the *Leishmania* species that causes VL in Latin America), which is highly conserved across *L. infantum* and *L. donovani*. The standard method for performing the rK39 enzyme-linked immunosorbent assay (ELISA) involves adding 50µl of a 1:100 dilution of the individuals’ sera to the wells of a micro-assay plate coated with rK39 antigen, incubating the plate with protein A-conjugated horseradish peroxidase to react with bound antibodies, and measuring the optical density (OD) of the sample relative to control sera to determine the antibody concentration [2]. Seropositivity is typically determined by a cut-off based on the distribution of ODs of non-endemic healthy controls, such as the mean OD plus two or three standard deviations.

**rK39 rapid diagnostic test (RDT)**

The rK39 immunochromatographic strip test (ICT) or rapid diagnostic test (RDT) is a dipstick version of the rK39 ELISA, developed for easier and cheaper use in the field. A finger prick of the patient’s serum or blood is added to the strip, on which there is a test line of fixed rK39 antigen and a red control line, and the strip is placed in a test tube with two or three drops of chase buffer solution. The sample solution migrates towards the test line, where it reacts within 10-20 minutes to produce a second red line next to the control line if antibodies to the rK39 antigen are present [3].

**Polymerase chain reaction (PCR) and quantitative PCR (qPCR)**

PCR and qPCR assays test for the presence of *L. donovani* DNA in clinical samples. Numerous protocols exist for PCR and qPCR assays (see [4] for a detailed review). Broadly speaking, PCR assays involve collecting a sample from the patient (splenic/bone marrow aspirate or whole blood/buffy coat/serum), pre-treating the sample to increase DNA yield (e.g. by incubating with proteinases and using lysis buffers), extracting the DNA (e.g. using phenol-chloroform or silica membrane), amplifying the DNA by PCR using primers that target specific *L. donovani* genes, and visualising the PCR amplification products on stained agarose gels [5–7]. In qPCR (or real-time PCR) the parasite load in the sample is quantified (in terms of equivalent number of parasites per ml of blood or μg of tissue DNA [8]) by adding a DNA probe/DNA-intercalating dye labelled with a fluorophore to the PCR mixture and measuring the fluorescence produced when it reacts with the amplified DNA at each PCR cycle (heating and cooling of the PCR mixture for amplification). The parasite load is then determined by comparison with a standard curve constructed from serial dilutions of parasite DNA of known concentration [8,9]. PCR and qPCR have high sensitivity and specificity for diagnosing clinical VL (over 90%) [10] and are reported to be able to detect parasite loads as low as 1 parasite/180μl blood [11]. Hence, they are believed to offer a marker for recent infection in healthy seronegative individuals [6].

**Leishmanin Skin Test (LST)**

The leishmanin skin test, also known as the Montenegro test, is a delayed-type hypersensitivity test, similar to the tuberculin skin test for TB. Leishmanin antigen is prepared from recently transformed promastigotes of a reference *Leishmania* strain (e.g. *L. donovani*, *L. infantum* or *L. major*) as a suspension of 5 x 10^6^ promastigotes/ml, and 0.1ml of the suspension is injected intradermally into the volar surface of the forearm [12]. After 48-72hrs, the induration at the injection site is measured in two perpendicular directions using the ball-point pen method [13], and if the mean of the two measurements is greater than 5mm the test is deemed positive. LST positivity is thought to represent durable cell-mediated immunity, which can last for several years or possibly for life [14,15]. The LST is generally negative in individuals with active VL due to anergy, and only becomes positive several months to years after successful treatment [16,17]. For individuals with no history of symptoms, a positive LST is thought to indicate previous asymptomatic infection and protection from future VL [12]. Thus, the proportion positive at a community level is presumed to represent the cumulative exposure of the community to the parasite.

**References**

1. Adams ER, Jacquet D, Schoone G, Gidwani K, Boelaert M. Leishmaniasis Direct Agglutination Test: Using Pictorials as Training Materials to Reduce Inter-Reader Variability and Improve Accuracy. PLoS Negl Trop Dis. 2012;6(12):1–6.

2. Burns JM, Shreffler WG, Benson DR, Ghalibt HW, Badaro R, Reed SG. Molecular characterization of a kinesin-related antigen of Leishmania chagasi that detects specific antibody in African and American visceral leishmaniasis. Proc Natl Acad Sci USA. 1993;90:775–9.

3. Chappuis F, Rijal S, Soto A, Menten J, Boelaert M. A meta-analysis of the diagnostic performance of the direct agglutination test and rK39 dipstick for visceral leishmaniasis. Br Med J. 2006;333(7571):723.

4. Reithinger R, Dujardin JC. Molecular diagnosis of leishmaniasis: Current status and future applications. J Clin Microbiol. 2007;45(1):21–5.

5. Alam MZ, Shamsuzzaman AKM, Kuhls K, Scho G. PCR diagnosis of visceral leishmaniasis in an endemic region, Mymensingh district, Bangladesh. Trop Med Int Heal. 2009;14(5):499–503.

6. Bhattarai NR, Auwera G Van Der, Khanal B, Doncker S De, Rijal S, Boelaert M, et al. PCR and direct agglutination as Leishmania infection markers among healthy Nepalese subjects living in areas endemic for Kala-Azar. Trop Med Int Heal. 2009;14(4):404–11.

7. Srivastava P, Gidwani K, Picado A, Van der Auwera G, Tiwary P, Ostyn B, et al. Molecular and serological markers of Leishmania donovani infection in healthy individuals from endemic areas of Bihar, India. Trop Med Int Heal. 2013;18(5):548–54.

8. Verma S, Kumar R, Katara GK, Singh LC, Negi NS, Ramesh V, et al. Quantification of parasite load in clinical samples of leishmaniasis patients: Il-10 level correlates with parasite load in visceral leishmaniasis. PLoS One. 2010;5(4).

9. Kaushal H, Bhattacharya SK, Verma S, Salotra P. Serological and Molecular Analysis of Leishmania Infection in Healthy Individuals from Two Districts of West Bengal, India, Endemic for Visceral Leishmaniasis. Am J Trop Med Hyg. 2017;96(6):1448–55.

10. de Ruiter CM, van der Veer C, Leeflang MMG, Deborggraeve S, Lucas C, Adams ER, et al. Molecular Tools for Diagnosis of Visceral Leishmaniasis: Systematic Review and Meta-Analysis of Diagnostic Test Accuracy. J Clin Microbiol. 2014;52(9):3147–55.

11. Deborggraeve S, Boelaert M, Rijal S, De Doncker S, Dujardin JC, Herdewijn P, et al. Diagnostic accuracy of a new Leishmania PCR for clinical visceral leishmaniasis in Nepal and its role in diagnosis of disease. Trop Med Int Heal. 2008;13(11):1378–83.

12. Bern C, Amann J, Haque R, Chowdhury R, Ali M, Kurkjian KM, et al. Loss of leishmanin skin test antigen sensitivity and potency in a longitudinal study of visceral leishmaniasis in Bangladesh. Am J Trop Med Hyg. 2006;75(4):744–8.

13. Sokal JE. Measurement of Delayed Skin-Test Responses. N Engl J Med. 1975;293(10):501–2. Available from: http://www.nejm.org/doi/abs/10.1056/NEJM197509042931013

14. Pampiglione S, Manson-Bahr PEC, La Placa M, Borgatti MA, Musumeci S. Studies in mediterranean leishmaniasis. 3. The leishmanin skin test in kala-azar. Trans R Soc Trop Med Hyg. 1975;69(1):60–8.

15. Nandy A, Neogy AB, Chowdhury AB. Leishmanin test survey in an endemic village of Indian kala-azar near Calcutta. Ann Trop Med Parasitol. 1987;81(6):693–9.

16. Haldar JP, Ghose S, Saha KC, Ghose AC. Cell-mediated immune response in Indian kala-azar and post-kala-azar dermal leishmaniasis. Infect Immun. 1983;42(2):702–7.

17. Neogy AB, Nandy A, Ghosh Dastidar B, Chowdhury AB. Leishmanin test in Indian kala-azar. Trans R Soc Trop Med Hyg. 1986;80(3):454–5.
