# Supplementary material for: Age trends in asymptomatic and symptomatic Leishmania donovani infection in the Indian subcontinent: A review and analysis of data from diagnostic and epidemiological studies
Source: PLoS Negl Trop Dis. 2018 Dec 6;12(12):e0006803. doi: 10.1371/journal.pntd.0006803 (PMC6283524; doi:10.1371/journal.pntd.0006803)
Supplement: S3 Data — Risk ratios (RRs) and odds ratios (ORs) for each study calculated relative to youngest age group. (DOCX) [file pntd.0006803.s008.docx]

**VL incidence studies**

Barnett et al, 2005 [1]

| Age group (yrs) | n | No. cases | Incidence/1000/yr (95% CI) | RR (95% CI) | p |
| --- | --- | --- | --- | --- | --- |
| 0-4 | 300 | 1 | 0.60 (0.02-3.71) | Ref. | - |
| 5-14 | 627 | 12 | 3.80 (1.98-6.69) | 5.74 (0.75-43.95) | 0.092 |
| 15-40 | 936 | 26 | 5.60 (3.63-8.14) | 8.33 (1.14-61.15) | 0.037 |
| ≥41 | 338 | 10 | 6.00 (2.84-10.88) | 8.88 (1.14-68.93) | 0.037 |

p = 0.87 for chi-squared test for trend in VL incidence for over-14s

Bern et al, 2005 [2]

| Age group (yrs) | n | No. cases | Incidence/1000/yr (95% CI) | RR (95% CI) | p |
| --- | --- | --- | --- | --- | --- |
| 0-4 | 333 | 22 | 12.01 (7.53-18.19) | Ref. | - |
| 5-14 | 683 | 71 | 18.90 (14.76-23.84) | 1.57 (0.99-2.49) | 0.053 |
| 15-24 | 450 | 41 | 16.57 (11.89-22.47) | 1.38 (0.84-2.27) | 0.206 |
| 25-34 | 336 | 27 | 14.61 (9.63-21.26) | 1.22 (0.71-2.09) | 0.479 |
| 35-44 | 253 | 13 | 9.34 (4.97-15.98) | 0.78 (0.40-1.51) | 0.459 |
| 45-54 | 172 | 5 | 5.29 (1.72-12.33) | 0.44 (0.17-1.14) | 0.091 |
| ≥55 | 174 | 3 | 3.13 (0.65-9.16) | 0.26 (0.08-0.86) | 0.027 |

p < 0.0001 for chi-squared test for trend in VL incidence for over-14s

Ferdousi et al, 2012 [3]

| Age group (yrs) | n | No. cases | Incidence/1000/yr (95% CI) | RR (95% CI) | p |
| --- | --- | --- | --- | --- | --- |
| 3-14 | 2344 | 111 | 15.78 (12.99-19.01) | Ref. | - |
| 15-45 | 3365 | 114 | 11.29 (9.32-13.57) | 0.72 (0.55-0.92) | 0.010 |
| ≥46 | 1052 | 23 | 7.29 (4.62-10.94) | 0.46 (0.30-0.72) | 0.001 |

p = 0.056 for chi-squared test for trend in VL incidence for over-14s

Hasker et al, 2012 [4]

| Age group (yrs) | n | No. cases | Incidence/1000/yr (95% CI) | RR (95% CI) | p |
| --- | --- | --- | --- | --- | --- |
| 0-4 | 12787 | 20 | 0.45 (0.27-0.69) | Ref. | - |
| 5-14 | 21020 | 79 | 1.07 (0.85-1.34) | 2.40 (1.47-3.92) | 0 |
| 15-24 | 14282 | 33 | 0.66 (0.45-0.93) | 1.48 (0.85-2.57) | 0.168 |
| 25-34 | 10993 | 31 | 0.81 (0.55-1.14) | 1.80 (1.03-3.16) | 0.04 |
| 35-44 | 8462 | 23 | 0.78 (0.49-1.17) | 1.74 (0.96-3.16) | 0.07 |
| ≥45 | 13666 | 21 | 0.44 (0.27-0.67) | 0.98 (0.53-1.81) | 0.955 |

p = 0.17 for chi-squared test for trend in VL incidence for over-14s

Hasker et al, 2013 [5]

| Age group (yrs) | n | No. cases | Incidence/1000/yr (95% CI) | RR (95% CI) | p |
| --- | --- | --- | --- | --- | --- |
| 2-9 | 3677 | 37 | 3.65 (2.57-5.03) | Ref. | - |
| 10-19 | 2909 | 20 | 2.49 (1.52-3.85) | 0.68 (0.40-1.17) | 0.168 |
| 20-29 | 1639 | 23 | 5.09 (3.23-7.64) | 1.39 (0.83-2.34) | 0.208 |
| 30-39 | 1554 | 17 | 3.97 (2.31-6.35) | 1.09 (0.61-1.92) | 0.774 |
| 40-49 | 1103 | 9 | 2.96 (1.35-5.62) | 0.81 (0.39-1.67) | 0.571 |
| 50-59 | 871 | 5 | 2.08 (0.68-4.86) | 0.57 (0.22-1.45) | 0.237 |
| 60-69 | 825 | 4 | 1.76 (0.48-4.50) | 0.48 (0.17-1.35) | 0.164 |
| ≥70 | 332 | 0 | 0.00 (0.00-4.03) | - | - |

p = 0.0013 for chi-squared test for trend in VL incidence for over-19s

Picado et al, 2014 [6]

| Age group (yrs) | n | No. cases | Incidence/1000/yr (95% CI) | RR (95% CI) | p |
| --- | --- | --- | --- | --- | --- |
| 0-6 | 1504 | 21 | 5.59 (3.46-8.54) | Ref. |  |
| 7-13 | 1869 | 22 | 4.71 (2.95-7.13) | 0.84 (0.47-1.53) | 0.573 |
| 14-24 | 981 | 19 | 7.75 (4.66-12.10) | 1.39 (0.75-2.57) | 0.297 |
| 25-39 | 1378 | 15 | 4.35 (2.44-7.18) | 0.78 (0.40-1.51) | 0.459 |
| ≥40 | 1806 | 18 | 3.99 (2.36-6.30) | 0.71 (0.38-1.33) | 0.291 |

p = 0.050 for chi-squared test for trend in VL incidence for over-13s

Singh et al, 2010 [7]

| Age group (yrs) | Incidence/1000/yr (95% CI) |
| --- | --- |
| 0-4 | 1.86 (0.89-3.90) |
| 5-14 | 8.32 (6.57-10.52) |
| 15-29 | 5.74 (4.32-7.63) |
| 30-44 | 6.05 (4.37-8.38) |
| 45-59 | 4.40 (2.56-7.56) |
| ≥60 | 2.30 (0.96-5.51) |

**Infection prevalence studies**

**DAT**

Hasker et al, 2013 [5]

| Age group (yrs) | n | No. positive | Prevalence (95% CI) | OR (95% CI) | p |
| --- | --- | --- | --- | --- | --- |
| 2-9 | 3858 | 100 | 0.026 (0.021-0.031) | Ref. | - |
| 10-19 | 2802 | 126 | 0.045 (0.038-0.053) | 1.77 (1.35-2.31) | <0.001 |
| 20-29 | 1565 | 92 | 0.059 (0.048-0.072) | 2.35 (1.76-3.14) | <0.001 |
| 30-39 | 1459 | 123 | 0.084 (0.071-0.100) | 3.46 (2.64-4.54) | <0.001 |
| 40-49 | 1021 | 101 | 0.099 (0.081-0.119) | 4.13 (3.10-5.49) | <0.001 |
| 50-59 | 812 | 87 | 0.107 (0.087-0.130) | 4.51 (3.35-6.08) | <0.001 |
| 60-69 | 767 | 97 | 0.126 (0.104-0.152) | 5.44 (4.07-7.28) | <0.001 |
| ≥70 | 321 | 51 | 0.159 (0.121-0.204) | 7.10 (4.96-10.17) | <0.001 |

Koirala et al, 2004 [8]

| Age group (yrs) | n | No. positive | Prevalence (95% CI) | OR (95% CI) | p |
| --- | --- | --- | --- | --- | --- |
| 0-1 | 25 | 0 | 0.000 (0-0.137) | - | - |
| 1-4 | 96 | 3 | 0.031 (0.006-0.089) | Ref. | - |
| 5-9 | 148 | 3 | 0.020 (0.004-0.058) | 0.64 (0.13-3.25) | 0.591 |
| 10-14 | 140 | 5 | 0.036 (0.012-0.081) | 1.15 (0.27-4.92) | 0.852 |
| 15-89 | 674 | 36 | 0.053 (0.038-0.073) | 1.75 (0.53-5.79) | 0.36 |

Ostyn et al, 2015 [9]

| Age group (yrs) | n | No. positive | Prevalence (95% CI) | OR (95% CI) | p |
| --- | --- | --- | --- | --- | --- |
| 2-5 | 30 | 2 | 0.067 (0.008-0.221) | Ref. | - |
| 6-15 | 128 | 11 | 0.086 (0.044-0.149) | 1.32 (0.28-6.28) | 0.73 |
| 16-25 | 72 | 7 | 0.097 (0.040-0.190) | 1.51 (0.29-7.72) | 0.622 |
| 26-35 | 55 | 7 | 0.127 (0.053-0.245) | 2.04 (0.40-10.52) | 0.393 |
| 36-45 | 35 | 6 | 0.171 (0.066-0.336) | 2.90 (0.54-15.58) | 0.215 |
| ≥46 | 98 | 7 | 0.071 (0.029-0.142) | 1.08 (0.21-5.48) | 0.929 |

Rijal et al, 2010 [10]

| Age group (yrs) | n | No. positive | Prevalence (95% CI) | OR (95% CI) | p |
| --- | --- | --- | --- | --- | --- |
| 2-6 | 677 | 18 | 0.027 (0.016-0.042) | Ref. | - |
| 7-13 | 1157 | 87 | 0.075 (0.061-0.092) | 2.98 (1.78-4.99) | <0.001 |
| 14-24 | 1109 | 87 | 0.078 (0.063-0.096) | 3.12 (1.86-5.23) | <0.001 |
| 25-39 | 1166 | 126 | 0.108 (0.091-0.127) | 4.44 (2.68-7.34) | <0.001 |
| ≥40 | 1288 | 171 | 0.133 (0.115-0.153) | 5.60 (3.42-9.20) | <0.001 |

Schenkel et al, 2006 [11]

| Age group (yrs) | n | No. positive | Prevalence (95% CI) | OR (95% CI) | p |
| --- | --- | --- | --- | --- | --- |
| 2-10 | 50 | 1 | 0.020 (0.001-0.106) | Ref. | - |
| 11-19 | 59 | 4 | 0.068 (0.019-0.165) | 3.56 (0.39-32.97) | 0.263 |
| 20-29 | 63 | 6 | 0.095 (0.036-0.196) | 5.16 (0.60-44.33) | 0.135 |
| 30-39 | 61 | 7 | 0.115 (0.047-0.222) | 6.35 (0.75-53.49) | 0.089 |
| 40-49 | 53 | 5 | 0.094 (0.031-0.207) | 5.10 (0.57-45.32) | 0.143 |
| 50-59 | 40 | 2 | 0.050 (0.006-0.169) | 2.58 (0.23-29.52) | 0.446 |
| 60-69 | 24 | 1 | 0.042 (0.001-0.211) | 2.13 (0.13-35.59) | 0.599 |
| 70-79 | 12 | 2 | 0.167 (0.021-0.484) | 9.80 (0.81-118.79) | 0.073 |
| 80-89 | 2 | 0 | 0.000 (0-0.842) | - | - |
| ≥90 | 1 | 0 | 0.000 (0-0.975) | - | - |

Singh et al, 2010 [12]

| Age group (yrs) | n | No. positive | Prevalence (95% CI) | OR (95% CI) | p |
| --- | --- | --- | --- | --- | --- |
| 2-6 | 1567 | 139 | 0.089 (0.075-0.104) | Ref. | - |
| 7-13 | 1930 | 342 | 0.177 (0.160-0.195) | 2.21 (1.79-2.73) | <0.001 |
| 14-24 | 1096 | 198 | 0.181 (0.158-0.205) | 2.27 (1.80-2.86) | <0.001 |
| 25-39 | 1484 | 300 | 0.202 (0.182-0.224) | 2.60 (2.10-3.23) | <0.001 |
| ≥40 | 1974 | 511 | 0.259 (0.240-0.279) | 3.59 (2.93-4.39) | <0.001 |

Topno et al, 2010 [13]

| Age group (yrs) | n | No. positive | Prevalence (95% CI) | OR (95% CI) | p |
| --- | --- | --- | --- | --- | --- |
| 0-4 | 27 | 2 | 0.074 (0.009-0.243) | Ref. | - |
| 5-14 | 117 | 9 | 0.077 (0.036-0.141) | 1.04 (0.21-5.12) | 0.96 |
| 15-29 | 79 | 12 | 0.152 (0.081-0.250) | 2.24 (0.47-10.72) | 0.313 |
| 30-44 | 57 | 10 | 0.175 (0.087-0.299) | 2.66 (0.54-13.09) | 0.229 |
| 45-59 | 60 | 6 | 0.100 (0.038-0.205) | 1.39 (0.26-7.37) | 0.7 |
| ≥60 | 15 | 0 | 0.000 (0-0.218) | - | - |

**rK39 ELISA**

Bern et al, 2007 [2002] [14]

| Age group (yrs) | n | No. positive | Prevalence (95% CI) | OR (95% CI) | p |
| --- | --- | --- | --- | --- | --- |
| 3-9 | 412 | 76 | 0.184 (0.148-0.225) | Ref. |  |
| 10-19 | 375 | 78 | 0.208 (0.168-0.253) | 1.16 (0.82-1.65) | 0.406 |
| 20-29 | 253 | 49 | 0.194 (0.147-0.248) | 1.06 (0.71-1.58) | 0.768 |
| 30-39 | 204 | 33 | 0.162 (0.114-0.220) | 0.85 (0.55-1.34) | 0.487 |
| 40-49 | 174 | 30 | 0.172 (0.119-0.237) | 0.92 (0.58-1.47) | 0.729 |
| 50-59 | 91 | 17 | 0.187 (0.113-0.282) | 1.02 (0.57-1.82) | 0.958 |
| ≥60 | 89 | 15 | 0.169 (0.098-0.263) | 0.90 (0.49-1.65) | 0.724 |

Bern et al, 2007 [2003] [14]

| Age group (yrs) | n | No. positive | Prevalence (95% CI) | OR (95% CI) | p |
| --- | --- | --- | --- | --- | --- |
| 3-9 | 456 | 63 | 0.138 (0.108-0.173) | Ref. |  |
| 10-19 | 434 | 73 | 0.168 (0.134-0.207) | 1.26 (0.87-1.82) | 0.214 |
| 20-29 | 332 | 62 | 0.187 (0.146-0.233) | 1.43 (0.98-2.10) | 0.066 |
| 30-39 | 231 | 33 | 0.143 (0.100-0.195) | 1.04 (0.66-1.64) | 0.867 |
| 40-49 | 185 | 28 | 0.151 (0.103-0.211) | 1.11 (0.69-1.80) | 0.665 |
| 50-59 | 96 | 8 | 0.083 (0.037-0.158) | 0.57 (0.26-1.23) | 0.149 |
| ≥60 | 93 | 7 | 0.075 (0.031-0.149) | 0.51 (0.22-1.15) | 0.103 |

Bern et al, 2007 [2004] [14]

| Age group (yrs) | n | No. positive | Prevalence (95% CI) | OR (95% CI) | p |
| --- | --- | --- | --- | --- | --- |
| 3-9 | 421 | 44 | 0.105 (0.077-0.138) | Ref. |  |
| 10-19 | 452 | 67 | 0.148 (0.117-0.184) | 1.49 (0.99-2.24) | 0.054 |
| 20-29 | 340 | 58 | 0.171 (0.132-0.215) | 1.76 (1.16-2.69) | 0.008 |
| 30-39 | 231 | 36 | 0.156 (0.112-0.209) | 1.58 (0.99-2.54) | 0.058 |
| 40-49 | 194 | 27 | 0.139 (0.094-0.196) | 1.39 (0.83-2.31) | 0.213 |
| 50-59 | 105 | 8 | 0.076 (0.033-0.145) | 0.71 (0.32-1.55) | 0.386 |
| ≥60 | 89 | 5 | 0.056 (0.018-0.126) | 0.51 (0.20-1.33) | 0.167 |

Hasker et al, 2013 [5]

| Age group (yrs) | n | No. positive | Prevalence (95% CI) | OR (95% CI) | p |
| --- | --- | --- | --- | --- | --- |
| 2-9 | 3858 | 81 | 0.021 (0.017-0.026) | Ref. |  |
| 10-19 | 2802 | 126 | 0.045 (0.038-0.053) | 2.20 (1.65-2.92) | <0.001 |
| 20-29 | 1565 | 95 | 0.061 (0.049-0.074) | 3.01 (2.23-4.08) | <0.001 |
| 30-39 | 1459 | 105 | 0.072 (0.059-0.086) | 3.62 (2.69-4.86) | <0.001 |
| 40-49 | 1021 | 96 | 0.094 (0.077-0.114) | 4.84 (3.57-6.56) | <0.001 |
| 50-59 | 812 | 96 | 0.118 (0.097-0.142) | 6.25 (4.60-8.49) | <0.001 |
| 60-69 | 767 | 106 | 0.138 (0.115-0.165) | 7.48 (5.54-10.10) | <0.001 |
| ≥70 | 321 | 37 | 0.115 (0.082-0.155) | 6.07 (4.04-9.13) | <0.001 |

**rK39 RDT**

Topno et al, 2010 [13]

| Age group (yrs) | n | No. positive | Prevalence (95% CI) | OR (95% CI) | p |
| --- | --- | --- | --- | --- | --- |
| 0-4 | 27 | 1 | 0.037 (0.001-0.190) | Ref. |  |
| 5-14 | 117 | 9 | 0.077 (0.036-0.141) | 2.17 (0.26-17.87) | 0.473 |
| 15-29 | 79 | 7 | 0.089 (0.036-0.174) | 2.53 (0.30-21.54) | 0.396 |
| 30-44 | 57 | 4 | 0.070 (0.019-0.170) | 1.96 (0.21-18.45) | 0.555 |
| 45-59 | 60 | 2 | 0.033 (0.004-0.115) | 0.90 (0.08-10.33) | 0.93 |
| ≥60 | 15 | 1 | 0.067 (0.002-0.319) | 1.86 (0.11-32.01) | 0.67 |

**PCR/qPCR**

Kaushal et al, 2017 [15]

| Age group (yrs) | n | No. positive | Prevalence (95% CI) | OR (95% CI) | p |
| --- | --- | --- | --- | --- | --- |
| 0-18 | 74 | 9 | 0.122 (0.057-0.218) | Ref. | - |
| 19-44 | 116 | 33 | 0.284 (0.205-0.376) | 2.87 (1.28-6.43) | 0.010 |
| ≥45 | 56 | 13 | 0.232 (0.130-0.364) | 2.18 (0.86-5.55) | 0.101 |

Topno et al, 2010 [13]

| Age group (yrs) | n | No. positive | Prevalence (95% CI) | OR (95% CI) | p |
| --- | --- | --- | --- | --- | --- |
| 0-4 | 27 | 3 | 0.111 (0.024-0.292) | Ref. | - |
| 5-14 | 117 | 10 | 0.085 (0.042-0.152) | 0.75 (0.19-2.92) | 0.676 |
| 15-29 | 79 | 6 | 0.076 (0.028-0.158) | 0.66 (0.15-2.83) | 0.574 |
| 30-44 | 57 | 6 | 0.105 (0.040-0.215) | 0.94 (0.22-4.09) | 0.936 |
| 45-59 | 60 | 3 | 0.050 (0.010-0.139) | 0.42 (0.08-2.24) | 0.31 |
| ≥60 | 15 | 0 | 0.000 (0-0.218) | - | - |

**LST**

Bern et al, 2006 [16]

| Age group (yrs) | n | No. positive | Prevalence (95% CI) | OR (95% CI) | p |
| --- | --- | --- | --- | --- | --- |
| 3-9 | 411 | 70 | 0.170 (0.135-0.210) | Ref. | - |
| 10-19 | 353 | 114 | 0.323 (0.274-0.374) | 2.32 (1.65-3.27) | <0.001 |
| 20-29 | 238 | 82 | 0.345 (0.284-0.409) | 2.56 (1.77-3.71) | <0.001 |
| 30-39 | 189 | 81 | 0.429 (0.357-0.502) | 3.65 (2.48-5.38) | <0.001 |
| 40-49 | 166 | 83 | 0.500 (0.422-0.578) | 4.87 (3.27-7.26) | <0.001 |
| 50-59 | 87 | 49 | 0.563 (0.453-0.669) | 6.28 (3.83-10.31) | <0.001 |
| ≥60 | 86 | 51 | 0.593 (0.482-0.698) | 7.10 (4.30-11.72) | <0.001 |

Nandy et al, 1987 [17]

| Age group (yrs) | n | No. positive | Prevalence (95% CI) | OR (95% CI) | p |
| --- | --- | --- | --- | --- | --- |
| 0-10 | 54 | 7 | 0.130 (0.054-0.249) | Ref. |  |
| 11-20 | 25 | 1 | 0.040 (0.001-0.204) | 0.28 (0.03-2.41) | 0.246 |
| 21-30 | 12 | 2 | 0.167 (0.021-0.484) | 1.34 (0.24-7.45) | 0.736 |
| 31-40 | 15 | 4 | 0.267 (0.078-0.551) | 2.44 (0.61-9.83) | 0.209 |
| 41-89 | 19 | 10 | 0.526 (0.289-0.756) | 7.46 (2.25-24.79) | 0.001 |

Patil et al, 2013 [18]

| Age group (yrs) | n | No. positive | Prevalence (95% CI) | OR (95% CI) | p |
| --- | --- | --- | --- | --- | --- |
| 1-10 | 36 | 7 | 0.194 (0.082-0.360) | Ref. | - |
| 11-25 | 18 | 11 | 0.611 (0.357-0.827) | 6.51 (1.85-22.88) | 0.003 |
| 26-40 | 33 | 22 | 0.667 (0.482-0.820) | 8.29 (2.76-24.84) | <0.001 |
| ≥40 | 11 | 4 | 0.364 (0.109-0.692) | 2.37 (0.54-10.40) | 0.254 |

Schenkel et al, 2006 [11]

| Age group (yrs) | n | No. positive | Prevalence (95% CI) | OR (95% CI) | p |
| --- | --- | --- | --- | --- | --- |
| 2-10 | 50 | 9 | 0.180 (0.086-0.314) | Ref. | - |
| 11-19 | 59 | 3 | 0.051 (0.011-0.141) | 0.24 (0.06-0.96) | 0.043 |
| 20-29 | 63 | 9 | 0.143 (0.067-0.254) | 0.76 (0.28-2.08) | 0.593 |
| 30-39 | 61 | 11 | 0.180 (0.094-0.300) | 1.00 (0.38-2.65) | 0.996 |
| 40-49 | 53 | 7 | 0.132 (0.055-0.253) | 0.69 (0.24-2.03) | 0.504 |
| 50-59 | 40 | 6 | 0.150 (0.057-0.298) | 0.80 (0.26-2.49) | 0.705 |
| 60-69 | 24 | 2 | 0.083 (0.010-0.270) | 0.41 (0.08-2.09) | 0.285 |
| 70-79 | 12 | 0 | 0.000 (0-0.265) | - | - |
| 80-89 | 2 | 1 | 0.500 (0.013-0.987) | 4.56 (0.26-79.88) | 0.299 |
| ≥90 | 1 | 0 | 0.000 (0-0.975) | - | - |

Yangzom et al, 2012 [19]

| Age group (yrs) | n | No. positive | Prevalence (95% CI) | OR (95% CI) | p |
| --- | --- | --- | --- | --- | --- |
| 2-15 | 108 | 7 | 0.065 (0.026-0.129) | Ref. |  |
| 16-45 | 201 | 23 | 0.114 (0.074-0.167) | 1.86 (0.77-4.50) | 0.166 |
| 46-89 | 87 | 13 | 0.149 (0.082-0.242) | 2.53 (0.96-6.66) | 0.059 |

**References**

1. Barnett PG, Singh SP, Bern C, Hightower AW, Sundar S. Virgin soil: the spread of visceral leishmaniasis into Uttar Pradesh, India. Am J Trop Med Hyg. 2005;73(4):720–5.

2. Bern C, Hightower AW, Chowdhury R, Ali M, Amann J, Wagatsuma Y, et al. Risk factors for kala-azar in Bangladesh. Emerg Infect Dis. 2005;11(5):655–62.

3. Ferdousi F, Alam MS, Hossain MS, Ma E, Itoh M, Mondal D, et al. Visceral Leishmaniasis Eradication is a Reality: Data from a Community-based Active Surveillance in Bangladesh. Trop Med Health. 2012;40(4):133–9.

4. Hasker E, Singh SP, Malaviya P, Picado A, Gidwani K, Singh RP, et al. Visceral Leishmaniasis, Rural Bihar, India. Emerg Infect Dis. 2012;18(10):1662–4. Available from: http://wwwnc.cdc.gov/eid/article/18/10/11-1083_article.htm

5. Hasker E, Kansal S, Malaviya P, Gidwani K, Picado A, Singh RP, et al. Latent infection with Leishmania donovani in highly endemic villages in Bihar, India. PLoS Negl Trop Dis. 2013;7(2):e2053.

6. Picado A, Ostyn B, Singh SP, Uranw S, Hasker E, Rijal S, et al. Risk factors for visceral leishmaniasis and asymptomatic Leishmania donovani infection in India and Nepal. PLoS One. 2014;9(1):1–8.

7. Singh VP, Ranjan A, Topno RK, Verma RB, Siddique NA, Ravidas VN, et al. Short Report: Estimation of Under-Reporting of Visceral Leishmaniasis Cases in Bihar, India. Am J Trop Med Hyg. 2010;82(1):9–11. Available from: http://www.ajtmh.org/cgi/doi/10.4269/ajtmh.2010.09-0235

8. Koirala S, Karki P, Das ML, Parija SC, Karki BMS. Epidemiological study of kala-azar by direct agglutination test in two rural communities of eastern Nepal. Trop Med Int Heal. 2004;9(4):533–7.

9. Ostyn B, Uranw S, Bhattarai NR, Das ML, Rai K, Tersago K, et al. Transmission of Leishmania donovani in the Hills of Eastern Nepal, an Outbreak Investigation in Okhaldhunga and Bhojpur Districts. PLoS Negl Trop Dis. 2015;9(8):e0003966. Available from: http://dx.plos.org/10.1371/journal.pntd.0003966

10. Rijal S, Uranw S, Chappuis F, Picado A, Khanal B, Paudel IS, et al. Epidemiology of Leishmania donovani infection in high-transmission foci in Nepal. Trop Med Int Heal. 2010;15(Suppl. 2):21–8.

11. Schenkel K, Rijal S, Koirala SS, Koirala SS, Vanlerberghe V, Van der Stuyft P, et al. Visceral leishmaniasis in southeastern Nepal: A cross-sectional survey on Leishmania donovani infection and its risk factors. Trop Med Int Heal. 2006;11(12):1792–9.

12. Singh SP, Picado A, Boelaert M, Gidwani K, Andersen EW, Ostyn B, et al. The epidemiology of Leishmania donovani infection in high transmission foci in India. Trop Med Int Heal. 2010;15(Suppl. 2):12–20.

13. Topno RK, Das VNR, Ranjan A, Pandey K, Singh D, Kumar N, et al. Asymptomatic infection with visceral leishmaniasis in a disease-endemic area in Bihar, India. Am J Trop Med Hyg. 2010;83(3):502–6.

14. Bern C, Haque R, Chowdhury R, Ali M, Kurkjian KM, Vaz L, et al. The epidemiology of visceral leishmaniasis and asymptomatic leishmanial infection in a highly endemic Bangladeshi village. Am J Trop Med Hyg. 2007;76(5):909–14.

15. Kaushal H, Bhattacharya SK, Verma S, Salotra P. Serological and Molecular Analysis of Leishmania Infection in Healthy Individuals from Two Districts of West Bengal, India, Endemic for Visceral Leishmaniasis. Am J Trop Med Hyg. 2017;96(6):1448–55.

16. Bern C, Amann J, Haque R, Chowdhury R, Ali M, Kurkjian KM, et al. Loss of leishmanin skin test antigen sensitivity and potency in a longitudinal study of visceral leishmaniasis in Bangladesh. Am J Trop Med Hyg. 2006;75(4):744–8.

17. Nandy A, Neogy AB, Chowdhury AB. Leishmanin test survey in an endemic village of Indian kala-azar near Calcutta. Ann Trop Med Parasitol. 1987;81(6):693–9.

18. Patil RR, Muliyil JP, Nandy A, Addy M, Maji A, Chatterjee P. Immuno-epidemiology of leishmanial infection among tribal population in kala-azar endemic areas: A community based study. Ann Trop Med Public Heal. 2013;6(1):50. Available from: http://www.atmph.org/text.asp?2013/6/1/50/115193

19. Yangzom T, Cruz I, Bern C, Argaw D, den Boer M, Vélez ID, et al. Endemic transmission of visceral leishmaniasis in Bhutan. Am J Trop Med Hyg. 2012;87(6):1028–37.
