# Supplementary material for: Correction: Age trends in asymptomatic and symptomatic Leishmania donovani infection in the Indian subcontinent: A review and analysis of data from diagnostic and epidemiological studies
Source: PLoS Negl Trop Dis. 2019 Feb 26;13(2):e0007150. doi: 10.1371/journal.pntd.0007150 (PMC6390991; doi:10.1371/journal.pntd.0007150)
Supplement: S1 File — (PDF) [file pntd.0007150.s001.pdf]

PLOS | NEGLECTED TROPICAL DISEASES

RESEARCH ARTICLE

Age trends in asymptomatic and symptomatic *Leishmania donovani* infection in the Indian subcontinent: A review and analysis of data from diagnostic and epidemiological studies

Lloyd A. C. Chapman[1,2,3]*, Alex L. K. Morgan[2,4], Emily R. Adams[5], Caryn Bern[6☯], Graham F. Medley[3☯], T. Déirdre Hollingsworth[1,2,7☯]

1 Zeeman Institute, University of Warwick, Coventry, United Kingdom, 2 School of Life Sciences, University of Warwick, Coventry, United Kingdom, 3 Centre for Mathematical Modelling of Infectious Diseases, London School of Hygiene and Tropical Medicine, London, United Kingdom, 4 School of Biological Sciences, University of Edinburgh, Edinburgh, United Kingdom, 5 Liverpool School of Tropical Medicine, Liverpool, United Kingdom, 6 Department of Epidemiology and Biostatistics, University of California San Francisco, San Francisco, California, United States of America, 7 Big Data Institute, Li Ka Shing Centre for Health Information and Discovery, University of Oxford, Oxford, United Kingdom

☯ These authors contributed equally to this work.
* Lloyd.Chapman@lshtm.ac.uk

Check for updates

OPEN ACCESS

Citation: Chapman LAC, Morgan ALK, Adams ER, Bern C, Medley GF, Hollingsworth TD (2018) Age trends in asymptomatic and symptomatic *Leishmania donovani* infection in the Indian subcontinent: A review and analysis of data from diagnostic and epidemiological studies. PLoS Negl Trop Dis 12(12): e0006803. https://doi.org/10.1371/journal.pntd.0006803

Editor: Genevieve Milon, Institut Pasteur, FRANCE

Received: March 30, 2018

Accepted: August 30, 2018

Published: December 6, 2018

Copyright: © 2018 Chapman et al. This is an open access article distributed under the terms of the Creative Commons Attribution License, which permits unrestricted use, distribution, and reproduction in any medium, provided the original author and source are credited.

Data Availability Statement: All relevant data are within the paper and its Supporting Information files.

Funding: This study was supported by the Bill and Melinda Gates Foundation (https://www.gatesfoundation.org/) in partnership with the Task Force for Global Health (https://www.taskforce.org/) through the NTD Modelling Consortium [OPP1053230] (LACC, GFM, TDH). The views, opinions, assumptions or any other information set

Abstract

Background

Age patterns in asymptomatic and symptomatic infection with *Leishmania donovani*, the causative agent of visceral leishmaniasis (VL) in the Indian subcontinent (ISC), are currently poorly understood. Age-stratified serology and infection incidence have been used to assess transmission levels of other diseases, which suggests that they may also be of use for monitoring and targeting control programmes to achieve elimination of VL and should be included in VL transmission dynamic models. We therefore analysed available age-stratified data on both disease incidence and prevalence of immune markers with the aim of collating the currently available data, estimating rates of infection, and informing modelling and future data collection.

Methodology/Principal findings

A systematic literature search yielded 13 infection prevalence and 7 VL incidence studies meeting the inclusion criteria. Statistical tests were performed to identify trends by age, and according to diagnostic cut-off. Simple reversible catalytic models with age-independent and age-dependent infection rates were fitted to the prevalence data to estimate infection and reversion rates, and to test different hypotheses about the origin of variation in these rates. Most of the studies showed an increase in infection prevalence with age: from ≲10% seroprevalence (<20% Leishmanin skin test (LST) positivity) for 0-10-year-olds to >10% seroprevalence (>20% LST-positivity) for 30-40-year-olds, but overall prevalence varied considerably between studies. VL incidence was lower amongst 0-5-year-olds than older age groups in most studies; most showing a peak in incidence between ages 5 and 20. The

out in this article are solely those of the authors and should not be attributed to the funders or any person connected with the funders. The funders had no role in study design, data collection and analysis, decision to publish, or preparation of the manuscript.

Competing interests: The authors have declared that no competing interests exist.

age-independent catalytic model provided the best overall fit to the infection prevalence data, but the estimated rates for the less parsimonious age-dependent model were much closer to estimates from longitudinal studies, suggesting that infection rates may increase with age.

Conclusions/Significance

Age patterns in asymptomatic infection prevalence and VL incidence in the ISC vary considerably with geographical location and time period. The increase in infection prevalence with age and peaked age-VL-incidence distribution may be due to lower exposure to infectious sandfly bites in young children, but also suggest that acquired immunity to the parasite increases with age. However, poor standardisation of serological tests makes it difficult to compare data from different studies and draw firm conclusions about drivers of variation in observed age patterns.

Author summary

As the elimination target for visceral leishmaniasis (VL) in the Indian subcontinent (<1 case/10,000 people/year) is approached, there is a growing need for surveillance tools with which to monitor transmission to ensure the target is sustained, especially given the large proportion of infections which are asymptomatic (~75–95%). One potential approach to estimate underlying transmission patterns may be to track age patterns in infection or cumulative exposure using diagnostic tests. However, current understanding of age patterns in asymptomatic infection and clinical VL is poor, in particular regarding possible age-dependence of infection rates. Our systematic review and pooled-analysis of age-stratified data on infection prevalence and disease incidence suggests that available diagnostics, as currently implemented, fail to meet the requirements for a reliable tool for assessing transmission, due to inconsistent standardisation and highly variable age-prevalence patterns across different settings. It also finds weak evidence for infection rates increasing with age, though further longitudinal studies are needed to test this hypothesis and to assess whether properly standardised diagnostic tests could be used to monitor ongoing transmission.

Introduction

The Indian subcontinent (ISC) appears to be on course to reach the target of elimination of visceral leishmaniasis (VL) as a public health problem (less than 1 case/10,000 people/year at sub-district level) in most sub-districts by 2020 [1–3]. Once this goal has been achieved, surveillance methods that require fewer resources than active case detection will be required to monitor transmission and provide early warning of possible resurgence [4]. One proposed method is monitoring age patterns in infection prevalence by serology or other diagnostic tests [4]. This approach has been used successfully for other vector-borne diseases, such as malaria [5–7], dengue [8] and Chagas disease [9–11], However, it is unclear whether it would be effective for VL surveillance with currently available diagnostics. Additionally, mathematical models of VL transmission dynamics are useful tools for understanding disease patterns and designing cost-effective control strategies [2,12–15], and it is unknown whether age-structure

is required within such models. The existence of evidence for age-related risk of infection and the viability of age-stratified serology as a post-elimination surveillance tool are the key issues we address in this review.

Almost 147 million people are at risk from VL in the ISC, caused by *Leishmania donovani* [16]. Elimination as a public health problem is considered to be possible based on the beliefs that indoor residual spraying (IRS) of insecticide is effective and that transmission is exclusively anthroponotic, combined with the availability of effective diagnostics and therapeutics to curtail the infectious period [17]. As of 2016, significant progress had been made towards reaching the elimination target, with an 82% decrease in new cases since 2011 and the target being reached in a number of previously endemic sub-districts [18]. In 2017, sub-district level incidence in India ranged between 0 and 12 cases/10,000 people/year and only 72 out of 633 endemic sub-districts were above the elimination target [19]; all endemic sub-districts in Bangladesh and districts in Nepal reported incidence $<$1 case/10,000 people/year [1,20].

Current elimination strategies in the ISC are based on early case detection and treatment, including education of at-risk populations, and methods to reduce abundance of the *Phlebotomus argentipes* sandfly vector such as IRS. Research has also identified the need to strengthen existing VL epidemiological surveillance programmes in order to aid disease detection and elimination [21]. However, only three large longitudinal VL studies have been carried out in order to assess infection and disease progression. These sources of data are the KALANET bed net trial in India and Nepal (2006–2009) [22], the Tropical Medicine Research Council (TMRC) study in India (2007-) [23], and the CDC- and ICDDR,B-funded study conducted by Bern and co-workers in Bangladesh (2002–2004) [24,25]. The scarcity of detailed, contemporary longitudinal data means that the progression, epidemiology, and transmission dynamics of the disease are still poorly understood [4].

The majority of infected individuals are asymptomatic, and never develop clinical symptoms, and estimates of the ratio of incident asymptomatic infection to incident VL vary from 4:1 [24] to 17:1 [26,27]. This variation may be related to differences in transmission intensity and levels of immunity in different regions and time periods (the ratio appears to decrease as VL incidence increases [27]), and/or differences in the definition of asymptomatic infection between studies (i.e. the diagnostic test(s) and cut-offs used), as there is no agreed definition or gold-standard test for asymptomatic infection. Changes in levels of immunity and spatial patterns of transmission over time will also have affected age distributions of infection and disease, so the age distributions may hold important information about transmission rates in different settings and how they have varied with time. Identification of the age groups with the highest prevalence of asymptomatic infection (who may act as a reservoir of transmission [28–30]) and those most at-risk of clinical VL could aid appropriate targeting of interventions to reduce transmission and disease.

Positivity on serological tests is an indication of exposure rather than immunity, but may be related to protective immunity. Age patterns in infection and disease are likely related to the immunological response to *L. donovani* infection, many aspects of which are still unknown. In particular, it is not known how long immunity to infection/disease lasts, the extent to which this depends on whether the individual recovered from asymptomatic infection or from clinical VL following treatment, and the degree of protection afforded [31]. Immunoglobulin G (IgG) antibody responses to *L. donovani* infection are not protective against disease [32–35], but cell-mediated immune responses are [36–40]. Thus, positivity on the leishmanin skin test (LST), a delayed-type hypersensitivity test, does represent protective immunity. Hence, assessing age patterns in sero-/LST-prevalence, sero-/LST-conversion and VL incidence may yield insights into durations of immune responses, and variation in immunity with age and VL endemicity.

For example, data on seroconversion incidence by age from the TMRC study [23] suggests that seroconversion rate may increase with age. If this is true, it has potentially important implications for control of VL and modelling of VL transmission, as it suggests that exposure to infected sandflies increases with age and/or that individuals living in endemic areas reconvert to seropositivity due to repeated bites from infected sandflies. Age-dependent exposure has so far only be included in one transmission model of VL [3,41]. Therefore, an important question for modelling is whether there is evidence of age-dependence in the infection rate in the infection age-prevalence data from other studies, and whether this effect should be included in transmission models.

In this paper, we review age-stratified data on *L. donovani* infection prevalence and clinical VL incidence and fit simple catalytic models to the age-prevalence data with the aim of improving understanding of age trends in asymptomatic and symptomatic infection. We estimate sero-, PCR- and LST- conversion and reversion rates from the data and compare them to estimates from longitudinal studies, and assess whether the conversion rates are age-dependent. Catalytic models have been used in meta-analyses for a number of diseases, including Chagas disease [11], malaria [6], varicella [42] and congenital rubella syndrome [43], to assess changes in transmission levels and identify shifts in infection prevalence towards older ages indicative of reduced transmission. Our goal is to provide insight into the epidemiology of VL and help inform improvements in interventions aimed at eliminating the disease from the ISC.

Methods

Systematic literature review

Relevant studies were identified through a systematic literature review. The search was conducted using the PubMed engine via the search terms set out in S1 Text. In addition to the PubMed search, the bibliographies of five reviews [4,27,44–46] relevant to visceral leishmaniasis incidence/infection prevalence were analysed for references eligible for this study. Only studies relevant to VL in the Indian sub-continent were included in the review. Any studies referring to cutaneous leishmaniasis, muco-cutaneous leishmaniasis or conducted in another geographical area were omitted.

The identified articles were subsequently screened based on their title and abstract, with eligible articles undergoing a full-text assessment. Articles were only included if age-stratified data was available for the incidence of clinical VL and/or prevalence of seropositivity/molecular test positivity/LST positivity, or the study-population/population-at-risk and the number of sero-/molecular-test-/LST-positives/clinical VL cases. All numerical data from the identified studies were doubly entered into spreadsheets and checked. The potential risk of bias in the included studies was assessed using the Newcastle-Ottawa bias assessment scale for observational studies [47] and the Cochrane risk of bias assessment tool for intervention studies [48].

The diagnostic tests used in the identified studies were: the direct agglutination test (DAT), recombinant K39 enzyme-linked immunosorbent assay (ELISA), rK39 rapid diagnostic test (RDT), polymerase chain reaction (PCR), quantitative PCR (qPCR), and the leismanin skin test (LST). Brief descriptions of the typical protocols for these tests are provided in S4 Text. The tests measure different aspects of infection and/or associated immune responses, as summarised in Table 1, so care is needed in interpreting and comparing their respective age prevalence patterns. DAT and rK39 ELISA are serological tests for antibodies (non-protective) against *L. donovani* parasites, the rK39 RDT is a rapid test form of the ELISA designed for diagnosis of clinical VL, PCR/qPCR is a molecular test for parasite DNA in the peripheral blood, and LST is a delayed-type hypersensitivity test for protective T-cell-mediated immune

Table 1. Characteristics of diagnostic tests used in identified studies.

Diagnostic test	DAT	rK39 ELISA	rK39 RDT	PCR/qPCR	LST
**Type**	Serological	Serological	Serological	Molecular	Skin
**Antigen used**	Whole-parasite lysate	Single recombinant K39	Single recombinant K39	N/A (test for parasite DNA)	Whole-parasite
**Biomarker measured**	IgG antibodies*	IgG antibodies*	IgG antibodies*	*L. donovani* DNA	T cells
**Marker of protective immunity**	x	x	x	N/A	✓
**Test for**	Recent infection/ asymptomatic infection	Recent infection/ asymptomatic infection	Clinical VL	Recent infection/ asymptomatic infection	Past infection
**Cut-off for positivity**	Sample dilution ratio at which agglutination still occurs. Variable between studies: ≥1:800–1:3200	Percentile (e.g. 99th), or mean + 2 or 3 standard deviations, of distribution of optical densities of samples from non-endemic healthy controls. Variable between studies.	Consistent for each manufacturer	Variable	≥5mm mean induration from LST injection
**Standardisation**	Variable	Variable	Consistent for each manufacturer	Variable	Consistent

* IgG = Immunoglobulin G

https://doi.org/10.1371/journal.pntd.0006803.t001

responses. Positivity on the different tests is believed to correlate to differing time since infection [23,49,50]. DAT and rK39 (ELISA and RDT) positivity represent more recent infection than LST positivity, since antibody responses are generally much shorter lived than cell-mediated immune responses [24,37,38,50,51]. The rK39 rapid test has the positive cut-off set for clinical diagnostic purposes and does not detect the low-titre antibody responses which are more common in asymptomatic infection. PCR positivity is also thought to represent recent infection [52], due to the ability of PCR to detect low numbers of parasites in peripheral blood during active infection, although longitudinal data on persistence of PCR positivity are lacking.

Although DAT and rK39 are both antibody tests, a number of important differences make it difficult to compare them, and to compare different DAT studies and different rK39 studies. These include the type of antigen used (single recombinant for rK39 vs whole-parasite lysate for DAT), how the tests are standardised against known positive and negative controls (which differs between studies), how the DAT is read [53] and the cut-off chosen for seropositivity (which also varies between studies). The molecular tests target different genes and are performed on varying quantities of blood, affecting the diagnostic accuracy.

Case definitions

Clinical VL. For the purposes of this review, incident cases of VL were defined as those who exhibited recognised clinical symptoms of VL (>2 weeks fever, swelling of the spleen and/or liver, weight loss, anaemia etc. [54]) and had a positive serological status, or individuals who were classified as 'clinical' or 'symptomatic' VL cases in their respective study (different studies use different terms), during the study period set out in the paper.

Asymptomatic infection. An asymptomatically infected individual was defined in this review as any individual who tested positive on a serological test for antibodies against *L. donovani* or a molecular test for *L. donovani* infection, and did not exhibit clinical symptoms. This definition covers those with active asymptomatic infection, i.e. those who harbour detectable numbers of live parasites, and those who may go on to develop clinical disease, and also

includes individuals who have recovered from infection who still possess antibodies against the parasite. Since most serological studies are unable to differentiate between seropositive individuals who have active infection and those that have dormant infection (are recovered), and do not distinguish pre-clinical infection from genuine asymptomatic infection, we do not differentiate these groups in this review.

Positive results by LST identify individuals with past infection, whether asymptomatic or symptomatic, who have developed a cell-mediated immune response. In longitudinal population-based data, positive LST results have been associated with ≥95% protection against subsequent clinical VL, and the prevalence in a population is an indication of herd immunity [38,39]. LST positivity is considered a marker of inactive disease and appears to be a long-lasting response, although probably not permanent in the absence of boosting. In communities with long-term endemic transmission, prevalence usually increases significantly with increasing age, reflecting cumulative incidence over time [38,39].

In addition, due to the scarcity of cross-sectional age-stratified asymptomatic infection prevalence data, we also include datasets with small numbers of clinical VL cases amongst the seropositive/LST-positive individuals. We highlight which datasets this applies to, and discuss the potential impact on the results, presenting the data on the prevalence of seropositivity/LST-positivity by VL status from the studies where available.

Data extraction

The following data was extracted from the papers: study/institution under which the data was collected (e.g. KALANET bed net trial, TMRC study, Indian Council of Medical Research); start & end date of the study; country, state, district & (where available) subdistrict of the study; number of villages; total population at risk; study population; number of excluded individuals; case definitions; type(s) of serological/diagnostic test used; age range & population of each age-group; number of individuals who underwent diagnostic testing, number of (sero) positive individuals; (sero)positive prevalence, number of clinical VL cases; and clinical VL incidence. Freely available software for digitising data [55] was employed to obtain data values from studies that did not provide numerical values for VL incidence/positive diagnostic prevalence, but provided relevant figures for calculation.

Statistical analysis

The age stratifications in the identified studies were used for statistical analyses (see S1 Data). For each diagnostic test, the prevalence of infection (the proportion of the population who were positive on the diagnostic test) in each age group in each study was plotted with exact binomial (Clopper-Pearson) 95% confidence intervals (CI) at the mid-point of the age group, to allow visual assessment and comparison of age trends in prevalence. Similarly, VL incidence (as number of cases/1000 study population/year) was calculated and plotted with 95% Poisson confidence intervals for each age group for each of the incidence studies. Odds ratios (ORs) for the risk of being seropositive/LST-positive and risk ratios (RRs) for having VL in each age group compared to the youngest age group were calculated with 95% CIs and 2-tailed p-values (using the Z-test) to identify any statistically significant variation in sero-/LST-positivity and VL incidence with age (at significance level 0.05). The chi-squared test for trend was used to assess trends in VL incidence with age (at significance level 0.05).

For studies in which multiple diagnostic tests were performed on the same individuals, the age-specific prevalence according to each test was plotted on the same graph for visual comparison and the non-parametric Friedman test was used to assess agreement in the age-specific prevalence between the different tests, using the VassarStats online computation tool [56].

Where possible, agreement between the different tests was also assessed by calculating Cohen's kappa coefficient [57] (a measure of agreement in classification between two tests, with 1 corresponding to perfect agreement and values ≤ 0 to no agreement), or retrieving the calculated value from the original study.

Catalytic modelling

The infection prevalence age distribution data was modelled using a reversible catalytic model [58] in which negative (sero-/LST-/PCR-negative) individuals become positive (sero-/LST-/PCR-positive) at a rate λ and revert back to sero-/LST-/PCR-negativity at a rate γ (see S2 Text for full details). We tested different versions of the model in which λ depends on age, the study (i.e. its location and time period) and/or the diagnostic test used and γ depends on the study and/or test (since the tests measure different immunological responses that may happen at different points after infection and over different timescales). We fitted the different versions of the model to the data to estimate λ and γ using maximum likelihood estimation and compared models using the Akaike information criterion (AIC) (see S2 Text for further details).

Results

Systematic literature review

A total of 19 age-stratified diagnostic and epidemiological studies that met the inclusion criteria were identified from the systematic literature review (Fig 1). Seven of these studies contained data on age-specific VL incidence (Table 2), and 13 studies contained age-stratified infection prevalence data (Table 3) ([23] contained both types of data). A further 8 studies containing age-stratified data are included in S1 Data (see S2 Data for definitions of variables in S1 Data), but are excluded from the analysis as the data is not comparable to that in the other studies, e.g. due to differences in study design or participant inclusion criteria. The assessment of the risk of bias in the included studies is shown in Tables 1–4 in S3 Text. Sources of potential bias include the lack of sample size justification in all but one of the cross-sectional studies and their failure to demonstrate comparability of non-respondents and comparability of different outcome groups. Loss to follow up was a potential source of bias in most cohort studies. We were unable to assess the risk of publication bias due to the small number of studies that met the inclusion criteria.

Age trends in VL incidence

The age-specific VL incidence curves for the studies in Table 2 are shown in Fig 2. The large variation in measured incidence between different studies is immediately apparent, and not unexpected given the differences in incidence between different geographical locations and different time periods. The highest average VL incidence, 13.2 cases/1000 people/year, was observed in the study of Bern et al [25], in Fulbaria upazila, Mymensingh district, Bangladesh, between 1999 and 2004.

There is a general pattern of decreasing VL incidence with increasing age beyond 20 years (Fig 2), although the trend is not significant for all studies (see p-values for chi-squared trend test in S3 Data). The exception (Barnett et al [59]) was conducted in outbreak villages in low-endemicity areas in Uttar Pradesh, India. Most of the studies for which incidence data for 0-10-year-olds is available show lower VL incidence among young children (0-5-year-olds) than older children and young adults (5-20-year-olds), with a peak in incidence in the 5-20yr age group (see RRs in S3 Data).

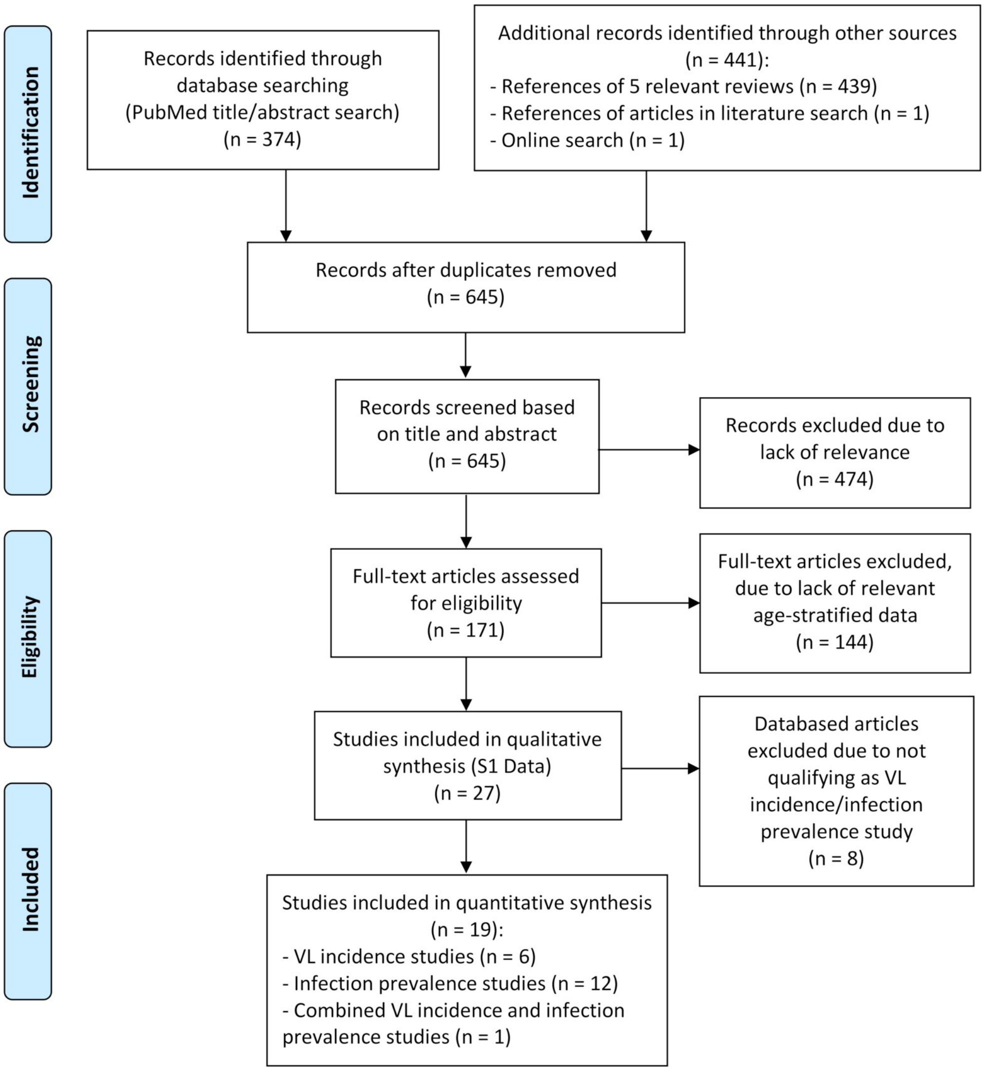

Fig 1. PRISMA flow diagram of the inclusion and exclusion of studies identified in the literature search.

https://doi.org/10.1371/journal.pntd.0006803.g001

Age trends in infection prevalence

Fig 3 shows age-prevalence curves for *L. donovani* infection from the studies in Table 3, with results separated by test. For studies in which multiple tests were performed on the population [23,24,68,70] the data are plotted together in Fig 4 to allow comparison of age-specific prevalence according to the different tests.

DAT. All studies that utilised DAT show a general increase in prevalence of DAT positivity with age up to 30-40yrs (Fig 3A). Above age 40, some studies show a continuing increase in seroprevalence with age [23,67,69] (see OR tables in S3 Data), while other studies suggest a decrease (albeit non-statistically significant) in DAT prevalence [66,68,70].

Since the DAT cut-off represents the dilution at which the sample is considered positive if it still reacts with the antigen, it might be expected that studies that used a lower DAT cut-off would generally report higher seroprevalences than those using a higher (more restrictive) cut-off. However, taking into account the variation in study settings, there is no clear trend in seroprevalence with DAT cut-off (Fig 3A), so differences in cut-off do not explain the variation in

Table 2. Summary of identified age-stratified clinical VL incidence studies.

Study	Location	Date		Study Population	Total VL Cases	Clinical VL Incidence (per 1000 people/yr)	Serological Test		
		From	To				rK39 RDT	rK39 ELISA	DAT
Barnett et al, 2005 [59]	Uttar Pradesh, India	1999	2004	2203	43	6.5	✓		
Bern et al, 2005 [25]	Bangladesh	01/1999	06/2004	2507*	182*	13.20	✓	✓	
Ferdousi et al, 2010 [60]	Bangladesh	08/2006	08/2008	6955	248	12.23	✓		
Hasker et al, 2012 [61]	Bihar, India	09/2008	10/2010	81210	207	0.73	✓		
Hasker et al, 2013 [23]	Bihar, India	03/2007	12/2009	19886	115	3.17		✓	✓
Picado et al, 2014 [62]	India + Nepal	11/2006	05/2009	17610	95	2.16	✓		✓
Singh et al, 2010a [63]**	Bihar, India	01/2006	12/2006	31324	177	5.7			

* Figures taken from Chapman et al (2015) [2].

** Information on serological testing performed for Singh et al, 2010a not available

https://doi.org/10.1371/journal.pntd.0006803.t002

Table 3. Summary of identified age-stratified infection prevalence studies.

Study	Location	Year	Total tested	Total positive	Prevalence (%)	Diagnostic Test				
						rK39 RDT	rK39 ELISA	DAT	LST	PCR/qPCR
Bern et al, 2006 [38]	Bangladesh	2002	1532	530	34.6				✓	
Bern et al, 2007 [24]*	Bangladesh	2002	1599	298	18.6		✓			
		2003	1827	274	15.0		✓			
		2004	1832	245	13.4		✓			
Hasker et al, 2013 [23]**	Bihar, India	2009	12605				✓	✓		
Kaushal et al, 2017 [64]	West Bengal, India	2014	246	55	22.4					✓
Koirala et al, 2004 [65]	Nepal	1996	1083	66	6.1			✓		
Nandy et al, 1987 [37]	West Bengal, India		125	24	19.2				✓	
Ostyn et al, 2015 [66]	Nepal	2014	418	40	9.6			✓		
Patil et al, 2013 [49]**	West Bengal, India	2000–2001			44.4				✓	
					44***			✓		
Rijal et al, 2010 [67]	Nepal	2006	5397	489	9.1			✓		
Schenkel et al, 2006 [68]**	Nepal	2003	365	28	7.5			✓		
			373	48	11.2				✓	
Singh et al, 2010b [69]	Bihar, India	2006	8051	1490	18.5			✓		
Topno et al, 2010 [70]**	Bihar, India		355	39	11.0			✓		
				28	7.9					✓
				24	6.8	✓				
Yangzom et al, 2012 [71]	Bhutan		396	43	10.9				✓	

* Age-stratified data previously unpublished.

** Denotes study where age stratified data was provided for each serological test.

*** DAT data not included in analysis since data deemed unreliable due to low cut-off used

https://doi.org/10.1371/journal.pntd.0006803.t003

Table 4. Infection prevalence by different diagnostics and VL status.

Study	Location	VL status	n	No, DAT/rK39 ELISA positive	No. LST positive	No. rK39 RDT positive	No. PCR/qPCR positive
Bern et al, 2007 [24]*	Bangladesh	Past VL	81	64 (79%)	51 (63%)		
		Current VL	14	14 (100%)	1 (7%)		
		Subsequent VL	39	14 (36%)	1 (3%)		
		No VL	1340	181 (14%)	452 (34%)		
Koirala et al, 2004 [65]	Bihar, India	Any	1083	66** (6.1%)			
Nandy et al, 1987 [37]	West Bengal, India	Past VL	25		20 (80%)		
		No h/o VL	125		24 (19%)		
Ostyn et al, 2015 [66]	Nepal	Past VL	23	22 (96%)			
		No VL	418	40 (9.6%)			
Rijal et al, 2010 [67]	Nepal	VL≤2yrs previously	93	90 (97%)			
		VL>2yrs previously	182	173 (95%)			
		No h/o VL	5120	226 (4.4%)			
Schenkel et al, 2006 [68]	Nepal	Past VL	19	18 (95%)	4 (21%)		
		No h/o VL	354	10 (2.8%)	44 (12%)		
Singh et al, 2010 [69]	Bihar, India	VL≤2yrs previously	269	250 (93%)			
		VL>2yrs previously	358	304 (85%)			
		No h/o VL	7418	936 (13%)			
Topno et al, 2010 [70]***	Bihar, India	Any	355	39**** (11%)		24† (7%)	28‡ (8%)

h/o = history of

* 2002 data

** Out of 66 DAT+: 22 current VL, 23 treated for VL≤1 year previously, 21 asymptomatic, of which 9 developed VL in ≤6mos

*** Out of 50 DAT+/rK39 RDT+/PCR+: 10 symptomatic (current VL / VL≤6mos previously), 40 asymptomatic, 2 of which had h/o VL≤6mos previously. Out of 38 asymptomatic without h/o VL, 7 developed VL in ≤6mos.

**** Out of 39 DAT+: 7 current VL, 2 with h/o VL≤6mos previously

† Out of 24 RDT+: 6 current VL, 2 with h/o VL≤6mos previously

‡ Out of 28 PCR+: 7 current VL, 1 with h/o VL≤6mos previously

https://doi.org/10.1371/journal.pntd.0006803.t004

seroprevalence between studies. Prevalence curves from Topno et al [70] (cut-off 1:800), Rijal et al [67], Hasker et al [23] (both cut-off 1:1600) and Koirala et al [65] (cut-off 1:2000) follow the expected trend, but those for the other studies do not. For example, the seroprevalence in the Schenkel et al [68] study (cut-off 1:3200), is higher than in the Hasker et al (cut-off 1:1600) and Koirala et al (cut-off 1:2000) studies up to age 50, and the seroprevalence in the Singh et al [69] study (cut-off 1:1600) is higher across all age groups than in all the other studies.

rK39 ELISA and RDT. Relatively few studies have examined age patterns in rK39 positivity, either by ELISA or the RDT. Those that have suggest rK39 seroprevalence increases up to at least age 25 (Fig 3B), with data from the TMRC study showing an increasing trend in rK39 ELISA positivity with age up to 70 ($p<0.001$ for chi-squared test for trend [23]). Differences in seroprevalence by rK39 ELISA between age groups in longitudinal data from the study of Bern et al [24] in Bangladesh from 2002 to 2004 were not statistically significant, apart from in the final year of the study where seroprevalence was higher amongst 10-39-year-olds than 3-9-year-olds (see S3 Data). The data also show that rK39 positivity decreased across all ages as the epidemic waned in the study population in 2003 and 2004 [51], towards a distribution

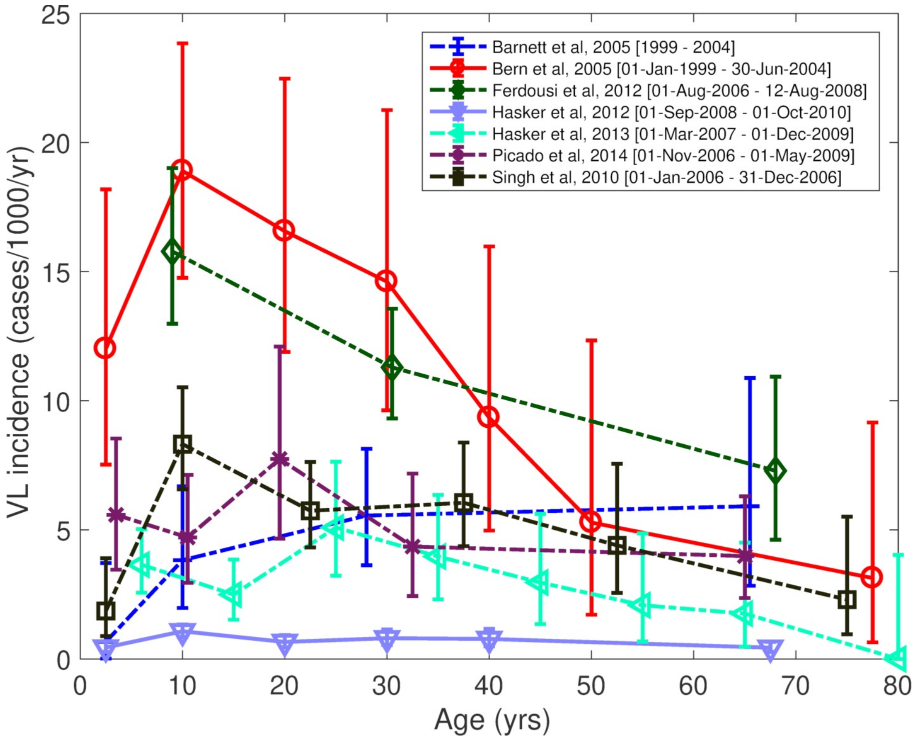

Fig 2. Age-specific visceral leishmaniasis incidence (cases per 1000 individuals per year) in different studies in the Indian subcontinent.

https://doi.org/10.1371/journal.pntd.0006803.g002

similar in shape to the cumulative VL incidence age distribution (Fig 2). The age-prevalence patterns in the TMRC and Bangladesh studies are not comparable due to differences in test standardisation and the cut-off used for asymptomatic seropositivity [23,51,72]. Similarly rK39 ELISA and rK39 RDT results are difficult to compare since positivity on the rK39 RDT generally corresponds to a higher rK39 antibody titre than is typically used as the rK39 ELISA seropositivity cut-off, particularly if the RDT is performed on whole blood rather than serum [73].

PCR/qPCR. Age-stratified data on prevalence of PCR positivity was available from only two studies [64,70] (Fig 3C). In the Topno et al study there was a decrease in the proportion PCR positive with age, although the trend was not significant, while in the Kaushal et al study the proportion qPCR positive was highest in the 19–44 age group (OR 2.87 (95% CI 1.28–6.43, p = 0.010) compared to the 0–18 age group). We note that PCR prevalence data for 668 individuals in the KALANET trial in India and Nepal also showed no consistent age pattern, with a slightly increasing prevalence with age in India and decreasing prevalence with age in Nepal [41].

Agreement between different tests. Despite the apparent agreement between the overall prevalence of seropositivity in each age group according to DAT and rK39 ELISA in the TMRC study (Fig 4B), agreement between the two tests was actually poor (Cohen's kappa, κ = 0.30), i.e. different individuals were seropositive according to the two tests. Poor agreement was also observed between rK39 RDT and qPCR results in the Kaushal et al [64] study (κ = 0.089). This may be due to a number of factors–differences in the sensitivities and specificities of the tests, differences in what the tests measure (as described above), and how well the tests were standardised and performed. The non-parametric Friedman test used to compare age-specific prevalence according to DAT, rK39 RDT and PCR for the Topno et al [70] (Fig 4D)

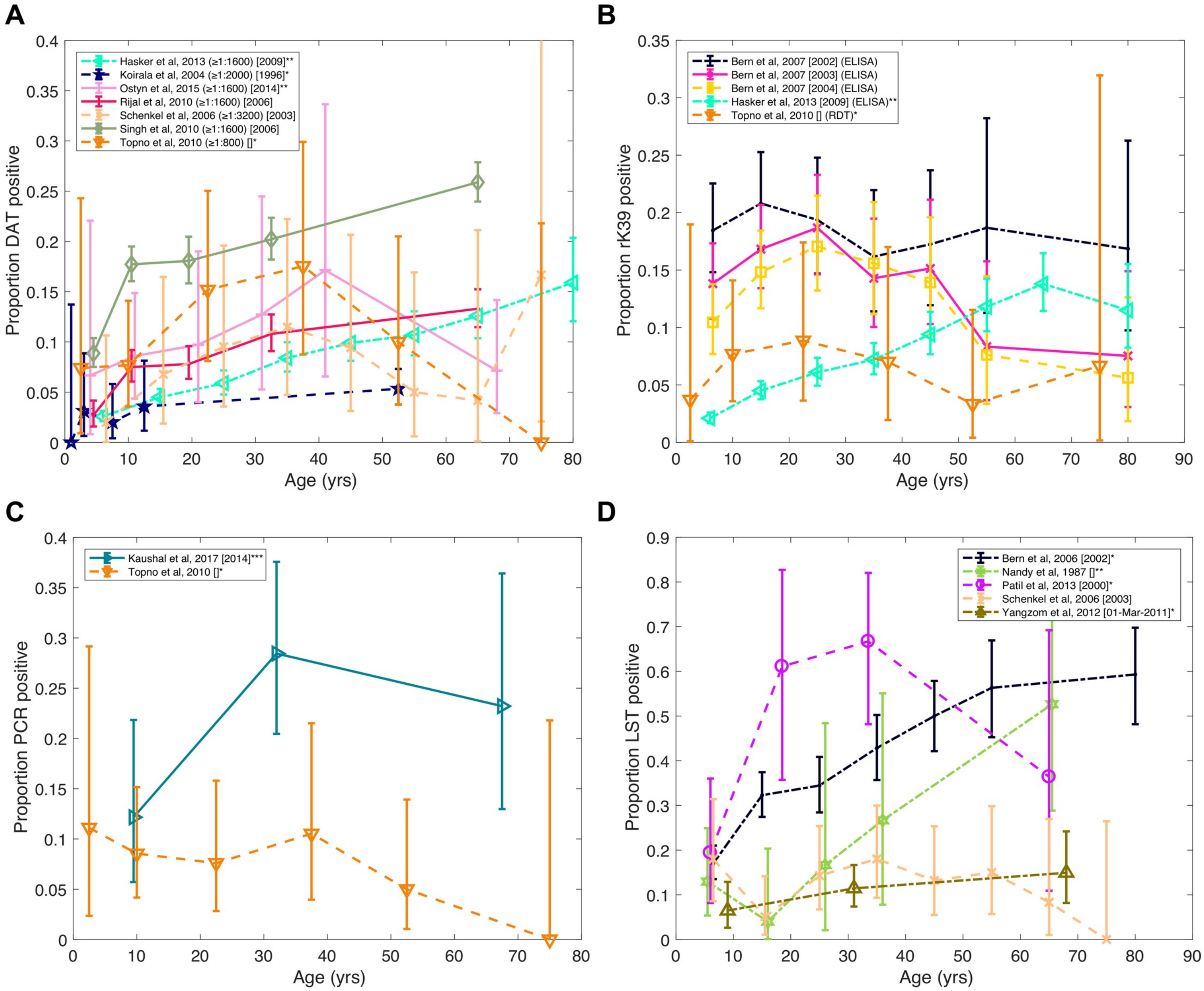

Fig 3. Age-prevalence distributions of positivity on different diagnostic tests for *Leishmania donovani* infection in the Indian subcontinent. (A) Seropositivity by Direct Agglutination Test (DAT) (numbers in parentheses denote cut-off for positivity), **(B)** seropositivity by rK39 Enzyme Linked Immunosorbent Assay (ELISA) or Rapid Diagnostic Test (RDT), **(C)** Polymerase Chain Reaction (PCR)/quantitative PCR (qPCR) positivity for parasite DNA, **(D)** LST positivity. All the prevalence studies include individuals with active or dormant asymptomatic infection, and some include a small number of active VL cases or exclude past VL cases. The year in which the survey was performed is shown in square brackets.* Prevalence includes a small number of active clinical VL cases.** Past VL cases excluded from prevalence.*** Data includes two individuals who were rK39 RDT+ but qPCR-.

https://doi.org/10.1371/journal.pntd.0006803.g003

study showed no significant difference in age-specific prevalence between the three tests ($p = 0.1969$). Furthermore, Cohen's kappa coefficients calculated for each pair of tests ranged from 0.56 (for DAT vs PCR) to 0.64 (for DAT vs rK39 RDT), indicating moderate to substantial pairwise agreement between the tests at individual-level [74] in this study.

LST. Age-prevalence patterns were not consistent across the five identified LST studies, and overall LST-positive prevalence varied considerably (from 10.9% to 44.4%) (Fig 3D). However, the studies of Bern et al [38] and Yangzom et al [71] showed clear increasing trends in the proportion positive with age, from 17% in the 3–9 age group up to 59% in the 60+ age

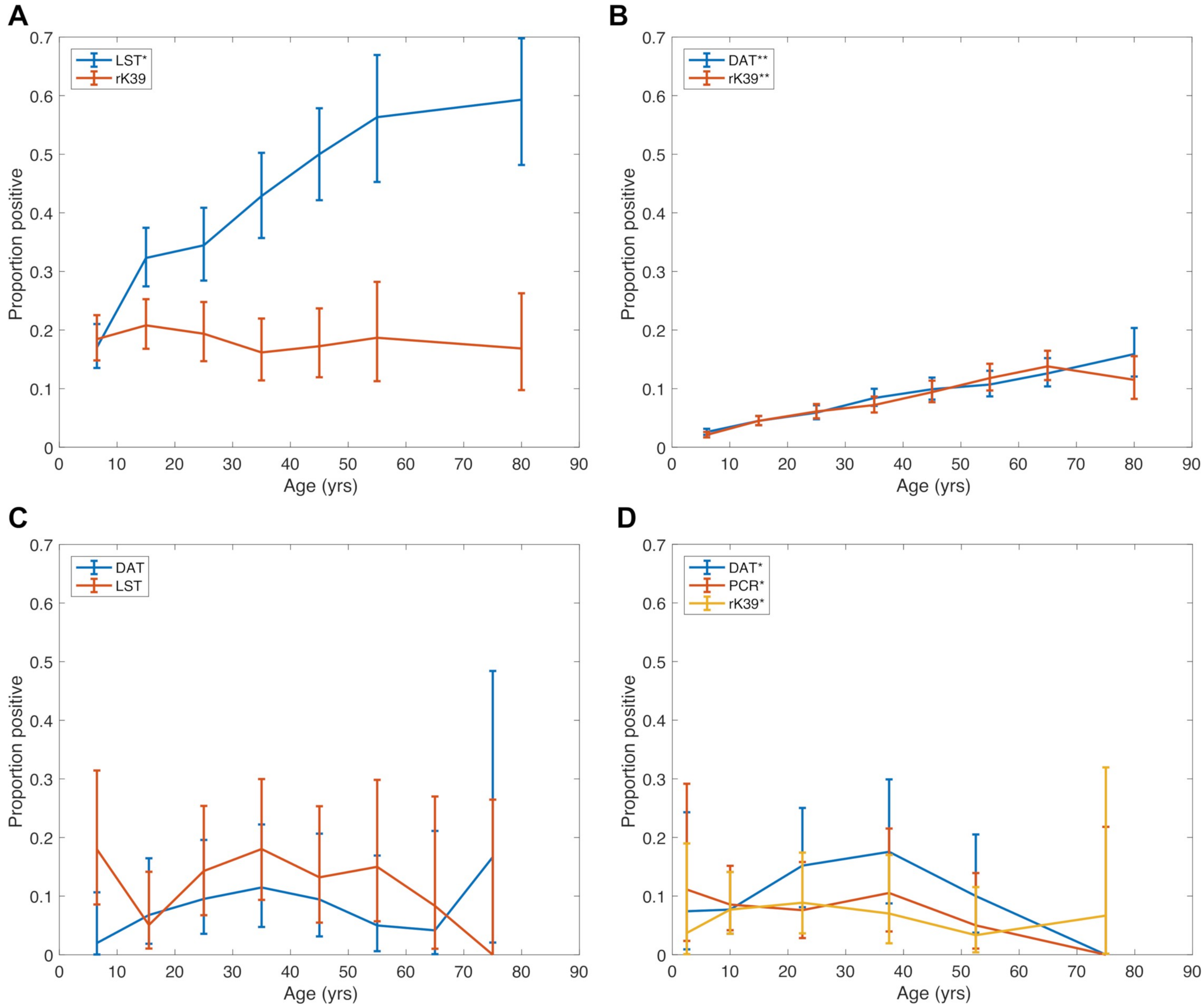

Fig 4. Comparison of age-prevalence distributions of positivity on different diagnostic tests for *L. donovani* infection in studies with multiple diagnostic tests. **(A)** Bern et al [24,38]: prevalences of rK39 ELISA and LST positivity, **(B)** Hasker et al [23]: rK39 ELISA and DAT seroprevalences **(C)** Schenkel et al [68]: DAT seroprevalence and LST positivity, **(D)** Topno et al [70]: DAT, rK39 RDT and PCR test positivities. * Prevalence includes a small number of active clinical VL cases.** Past VL cases excluded from prevalence.

https://doi.org/10.1371/journal.pntd.0006803.g004

group in the former, and from 6.5% in the 2–15 age group to 15% in over-45s in the latter, despite being performed in settings with very different endemicities.

The highest overall LST-positive prevalence (44%) was in the study of Patil et al [49] in West Bengal. In this study, a higher proportion of young and middle-aged adults (aged 11 to 40) than 0-10-year-olds and over-40s were LST positive. DAT data from the same individuals showed higher seroprevalence among 11-40-year-olds (48%) than 1-10-yr-olds and over-40s (40% and 41% respectively), suggesting higher rates of relatively recent infection and consequent LST conversion in this age group.

Similarly to Bern et al's study, the study of Nandy et al [37] in an endemic village near West Bengal showed a general increase in prevalence of LST positivity with age, albeit with a higher prevalence among 0-10-year-olds than 11-20-year-olds.

In contrast to the other studies, the prevalence of LST positivity in the Schenkel et al [68] study in southeastern Nepal was relatively low across all age groups (0–18%) and showed no pattern with age. It is possible that a smaller proportion of this population was exposed to the parasite and developed cell-mediated immunity due to the lower endemicity of the study location, but issues with the sensitivity and potency of the *L. infantum* antigen used [38] likely contributed to the low LST prevalence observed, especially given the low proportion of LST positivity in past VL cases (21% compared to 63% and 80% in the Bern and Nandy studies). These data suggest that standardisation of leishmanin is critical, and was not uniform, across these studies.

Effect of VL status on diagnostic results. Since the infection prevalence studies shown in Fig 3 differ in terms of whether they include/exclude active/past VL cases, and this may affect the age-prevalence distribution, we also recorded data on prevalence of sero-/LST-positivity by VL status from these studies when available (see Table 4). The high prevalences of DAT/rK39 ELISA positivity amongst past VL cases shown in Table 4 reflect the persistence of high antibody levels in treated VL patients [23,75], and are likely to have affected the age-prevalence distributions to some extent since they are much higher than the prevalences in individuals with no history of VL. Likewise, prevalence of LST positivity was much higher in past VL cases than in individuals without previous VL, suggesting that inclusion of past VL cases will have affected the age-prevalence pattern of LST positivity. However, the number of *active* VL cases observed in any of the cross-sectional studies was small relative to the number of sero-/LST-positive individuals, so their inclusion is unlikely to have significantly biased the age-prevalence distributions.

Catalytic modelling. The best-fitting model based on AIC is the model in which the conversion and reversion rates, λ and γ, are independent of age but study-specific. This has a lower AIC than the model with age-dependent and study-specific conversion rates and study-specific reversion rates (ΔAIC = 6.5), which is the next best-fitting model (and has a higher likelihood). The models in which the conversion and/or reversion rates are test-specific or the same across all studies have considerably higher AICs (see S2 Text for full results). The (λ,γ) estimates for the age-independent and age-dependent models with study-specific conversion and reversion rates are shown in Fig 5, along with estimates of seroconversion and seroreversion rates from studies in which there were repeated measurements [23,24,50]. Whilst there appears to be some clustering in the estimated rates by test type in the age-independent model (Fig 5A)–e.g. the LST reversion rates are generally lower than the DAT reversion rates, which are generally lower than the rK39 reversion rates–this accounts for less of the variation in the estimates than the data source study. The ranges of the estimated λ and γ values for each test are large, e.g. [0.0037,0.026]yr^{-1} and [0.021,0.18]yr^{-1} respectively for DAT, and the median values for DAT, $\lambda = 0.0091yr^{-1}$ and $\gamma = 0.077yr^{-1}$, correspond to 20% of individuals having been infected by age 24 and an average duration of positivity of 13yrs. Many of the estimated conversion rates and nearly all of the estimated reversion rates are lower than the estimates from the longitudinal studies, e.g. for the Hasker et al 2013 study the estimated rates for DAT are $\lambda = 0.0037yr^{-1}$ and $\gamma = 0.021yr^{-1}$, while the longitudinal estimates are $\lambda = 0.026yr^{-1}$ and $\gamma = 0.46yr^{-1}$. Two possible explanations for this are that the infection rate does actually increase with age, and/or that the infection rate is highly heterogeneous, i.e. transmission is localised around infectious individuals in such a way that only a certain proportion of the population (corresponding to the maximum in the age-prevalence curve) ever becomes infected. Accounting for this age-dependence and/or spatial heterogeneity would lead to higher estimates for λ

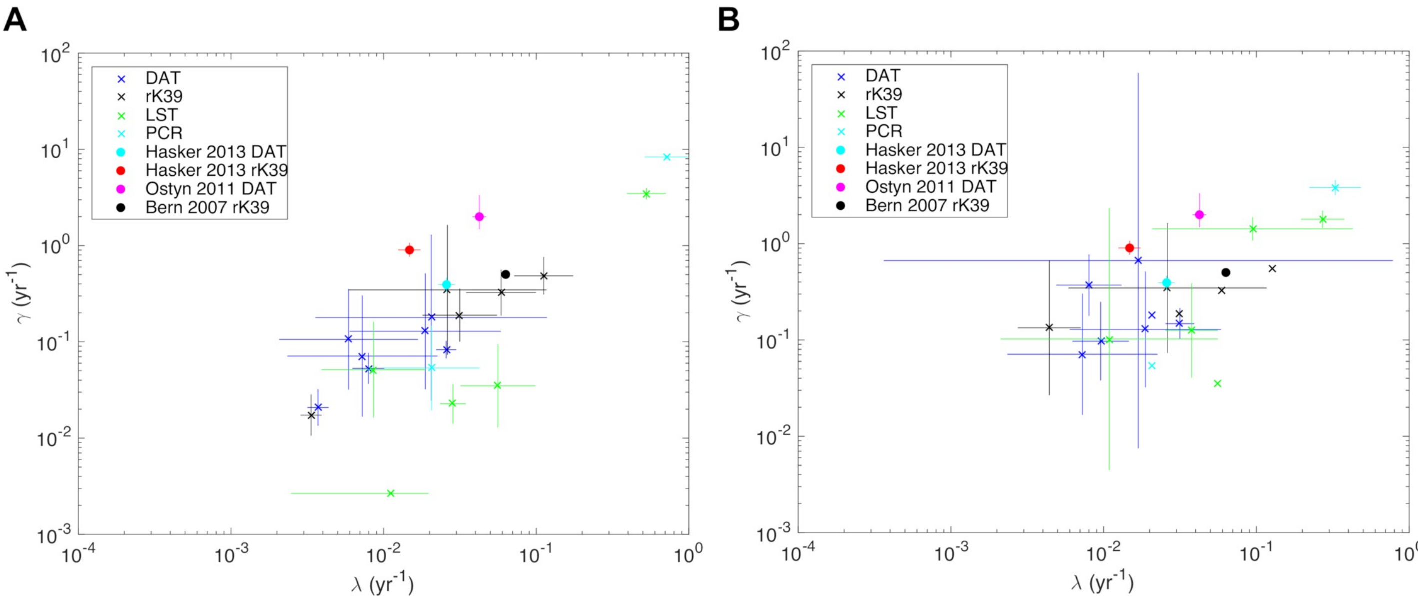

Fig 5. Estimated conversion and reversion rates, λ and γ, from (A) age-independent ($\lambda = b_0$ = constant) and (B) age-dependent ($\lambda = b_0 + b_1 \times$ age) reversible catalytic models for the age-prevalence distributions of infection for the different studies in Table 3. Crosses show the maximum likelihood estimates for (λ,γ) for each study, dots (λ,γ) estimates from longitudinal studies [23,24,50], and lines their 95% confidence intervals. λ estimates in (B) are at age 20yrs (i.e. $\lambda = b_0 + 20b_1$).

https://doi.org/10.1371/journal.pntd.0006803.g005

and γ, since if λ increases with age a higher value of γ is required to achieve the same prevalence, and if transmission decreases with distance from infected individuals a higher average value of λ is required to reach the same prevalence (and therefore a higher value of γ to obtain the same age-prevalence distribution). The estimated rates from the age-dependent model are indeed much closer to the longitudinal estimates, e.g. for the Hasker et al 2013 study $\lambda = 0.022\text{yr}^{-1}$ at the median age (19yrs) and $\gamma = 0.37\text{yr}^{-1}$ (corresponding to an average duration of positivity of 2.7yrs), which supports the theory that λ is age-dependent. However, it is not possible to assess the impact of spatial heterogeneity in transmission from the available data.

Discussion

The systematic review and meta-analysis of the age-related prevalence and incidence of *L. donovani* infection and disease in the ISC in this paper has found weak evidence for age-related infection rates. However, there is considerable variation in the data that might be masking such an effect, so no definitive conclusion can be drawn. Nevertheless, it seems likely that even if there is an age-effect in exposure and infection, it is less significant than the strong temporal and spatial dependence of VL transmission risk [18].

There is considerable variation in observed *L. donovani* infection prevalence and disease incidence by geographical location, endemicity level and time period. Broadly speaking, studies performed more recently have found lower VL incidence rates due to the significant overall decline in VL incidence in India, Bangladesh and Nepal [76] over the past decade, and those performed in India and Bangladesh have observed higher VL incidence and infection prevalence than those in Nepal (see Tables 2 and 3, Table S1 in [50], and Table J S1 text in [27]).

Despite this variation, there is a relatively consistent pattern of lower seroprevalence (by DAT and rK39) and VL incidence among young children (0-5-year-olds) than older children (5-20-year-olds). Various theories have been put forward to explain the lower reported VL incidence among young children [77], including lower exposure to bites from infected sandflies in young children, possibly based on different sleeping behaviours.

Reported age patterns in seroprevalence are less consistent over age 20yrs and vary by serological test. Some DAT studies suggest continually increasing seroprevalence with age, but other DAT studies and rK39 ELISA surveys suggest lower or similar seroprevalence for over-40s as for 20-40-year-olds. One potential explanation is differences in boosting of antibody levels in asymptomatically infected individuals from repeated exposure to the parasite from infected sandflies [23]. This theory is supported by the increase in both the DAT seroprevalence and DAT/rK39 seroconversion rate with age observed in highly endemic villages in Muzaffarpur, Bihar, in the TMRC study [23].

Prevalence of LST positivity appears to generally increase with age in highly endemic settings, likely due to an increase in cumulative exposure to the parasite with age and the potentially long duration of cell-mediated immunity. Data from LST surveys in Ethiopia conducted one year apart showed 5% reversion from positive to negative LST [78] suggesting that repeated exposure to infected sandflies may be required to maintain LST positivity; this may help explain some of the differences in age patterns between the studies. Another cause of differences may be the leishmanin antigen (*L. major* or *L. infantum*), and its sensitivity and potency [38]. Although LST is a potentially valuable tool for assessing transmission intensity and immune status in communities [37,78], new sources of well-standardised leishmanin antigen are required, preferably produced under internationally recognized Good Manufacturing Practices (GMP). The only antigen currently available (*L. major* MRHO/IR/75/ER antigen from the Pasteur Institute in Iran) gave unreliable results when it was trialled for use in the KALANET study and was abandoned [79].

Nearly all of the reviewed VL incidence studies showed a peak in incidence between the ages of 5 and 20, with lower incidence in adults over 40. Together with the apparent general increase in seroprevalence and LST positivity with age, this suggests that immunity to VL increases with age. However, VL incidence varies in long-term cycles at district and state level in the ISC with peaks approximately 15 years apart [80–82], so age patterns of infection prevalence and cumulative VL incidence will vary according to the point in these epidemic cycles at which the studies were conducted. Likewise, within these longer term cycles VL tends to occur in localised epidemics, affecting villages for a few years before moving to neighbouring villages, so the timing and location of studies relative to these micro-epidemics will have an impact on age patterns [50,83]. There is also seasonality in VL incidence due to seasonality in sandfly abundance [3,77,84] and evidence of seasonal variation in seroprevalence [85]. Thus, the time of year at which the cross-sectional serological surveys were performed and period over which the cumulative VL incidence was recorded may have also affected the observed age patterns.

Accurate comparison of age trends in asymptomatic infection and VL incidence between studies is hindered by a lack of consistency in study design, data presentation, and standardisation of diagnostic test protocols and cut-offs for positivity. Diagnostic studies vary regarding inclusion/exclusion of active/past VL cases, despite the fact that this can affect the age pattern of infection (Table 4 and S1 Data). Many serological and epidemiological studies do not report the age distribution of seropositivity or VL incidence, or for the latter only report the age distribution of cases and not that of the whole study population, even though this data is routinely gathered and potentially epidemiologically useful. Thus, there is relatively little published data with which to assess age patterns in infection prevalence and disease incidence and how they relate to each other. More regular and consistent collection and presentation of age prevalence and incidence data would greatly assist in identification and comparison of age trends in asymptomatic infection and clinical VL.

Given issues of different test standardisation between studies, potential difficulties with standardising test protocols across studies in the future, and possible repeated sero- and LST-conversion from re-exposure, data from longitudinal studies on sero- and LST- conversion

and reversion may be more useful than data from cross-sectional surveys at present. Comparatively few longitudinal studies of sero- or LST- conversion and progression to VL associated with different diagnostic results have been conducted. Those that have been performed suggest that agreement between different diagnostics in terms of predicting risk of progression to VL is poor [26]. What is known is that high-titre DAT/rK39 ELISA seropositivity and seroconversion correlate with progression to VL [26,51], and LST positivity means a very low risk of subsequent VL in the absence of immunosuppression [38,78,86].

Although we found no statistically significant differences in age-specific infection prevalence according to different diagnostics tested on the same study population, agreement between different diagnostics in these studies was generally poor, as found in other studies [52,87–89]. A number of authors have suggested that these discrepancies are due to differences in kinetics between bio-markers and the sensitivities of different diagnostics, such that different diagnostics become positive/negative at different points in the course of infection and to varying degrees dependent on whether the individual is asymptomatic or pre-symptomatic [23,49,50,52]. Mathematical modelling studies have used combinations of different diagnostic test results to try to stage infection [41,51,90]. However, the true picture is likely more complicated, due to individual-level heterogeneity in parasite loads and immune responses, potential boosting of antibody levels in asymptomatically infected individuals from re-exposure and intermittent proliferation of parasites contained in safe target cells [23], and the unknown temporal dynamics of different infection markers in asymptomatic infection. Various studies have investigated the temporal dynamics of different biomarkers in clinical VL [23,36,75,91–93], mostly from treatment onwards, but equivalent studies for asymptomatic infection are lacking [50], no doubt in part due to the difficulty of defining asymptomatic infection.

The parameter estimates obtained from the catalytic model reflect the significant variation in infection and reversion rates by study location and time period. There is more variation between studies than between tests. The catalytic models with an age-independent conversion rate give underestimates of the conversion and reversion rates based on direct estimates from longitudinal data. Models with an increase in the conversion rate with age provide estimates closer to the longitudinal estimates, suggesting that age-dependent exposure or age-dependent seroconversion upon infection are possible explanations for the discrepancy. However, evidence that the conversion rate increases with age is relatively limited, and time-dependent changes will induce age-dependent patterns for long duration markers. For example, a seroconversion rate that increases linearly with age could also be the result of a linear reduction in transmission intensity with time. The trend in the Hasker et al study [23] is clear, but overall the age-dependent catalytic models do not fit the data better than the age-independent models, and other studies do not show the same trend in seroconversion rate with age [24,62]. Nevertheless, the lack of strong evidence that the infection rate is age-independent suggests that age-dependence should be included in models of VL transmission dynamics in the ISC. If exposure is not age-dependent, a natural question is why there is an age pattern in VL incidence. The simplest explanation is that risk of disease given infection increases with age up to 15–20 years and then decreases. Another possible explanation for the estimated conversion and reversion rates being lower than expected is that infection and immunity are highly localised, and so only a certain proportion of the study population is actually at risk of infection, rather than the whole study population being equally at risk as assumed in the analysis.

Due to a lack of available disaggregated data, we have not explored the role of possible confounders for age patterns in seroprevalence and VL incidence, such as sex and spatial proximity to VL cases and infected individuals. Various studies have observed higher VL incidence in males than females [62,77,81,94,95] and some have observed higher sero-prevalence/conversion in males [62,67,69], both of which may be caused by socio-behavioural and/or biological

factors [67,69,95–99]. The differences between sexes appear to vary with age [67,69,77], so may affect observed age patterns. Proximity to VL cases and/or infected individuals is known to be a risk factor for infection and disease, with numerous studies showing higher rates of seropositivity, seroconversion, VL, and LST positivity in individuals who share a house with or live near a previous VL case or seropositive individuals [24,25,37,62,67,87,88]. Although we excluded any studies that focused on close contacts of VL cases, differences in spatial clustering of transmission in different settings may have affected the observed age patterns in seroprevalence and VL incidence. Due to the absence of published age-stratified data on other potentially promising diagnostics, such as urinary antigen tests, sandfly saliva antibody tests, and interferon gamma release assays [4,100,101], we were only able to review age patterns for the most widely used diagnostics.

Conclusion

The main conclusion that can be drawn from this review is that age patterns of *L. donovani* infection measured using current serological tests appear to be too variable across different settings and endemicity levels to be used to monitor levels of ongoing transmission post elimination. The extent to which this variability is due to genuine variation in the age-prevalence distribution with location and time, to properties of the tests, and/or to inconsistent test standardisation is unclear. However, the fact that significant age trends have only been observed in large studies suggests that very large sample sizes would be needed to reliably detect changes in transmission levels based on age patterns. Further longitudinal studies are required to improve understanding of the dynamics of serological responses and to determine whether serological tests can be used as a surveillance tool to monitor transmission. If such studies can demonstrate that well-standardised serological testing provides a reliable indicator of transmission, cross-sectional serological surveys may still prove to be a useful tool for achieving and sustaining elimination of VL as a public health problem in the ISC.

Supporting information

S1 Checklist. PRISMA checklist. Checklist for reporting of systematic reviews and meta-analyses.
(DOC)

S1 Text. Details of literature search including search terms and reviews used.
(DOCX)

S2 Text. Further details on the catalytic modelling of the infection prevalence data. Model fitting and comparison, and parameter estimates.
(PDF)

S3 Text. Assessment of potential risk of bias in included studies.
(DOCX)

S4 Text. Descriptions of the diagnostic tests used in the identified studies.
(DOCX)

S1 Data. Age stratified data on clinical VL incidence and infection prevalence. Spreadsheet includes data from the 19 studies included in the review and age-stratified data from 8 studies that did not meet the eligbility criteria.
(XLSX)

S2 Data. Metadata for S1 Data. Definitions of variables.
(XLSX)

S3 Data. Risk ratios for VL and odds ratios for diagnostic test positivity by age group for the reviewed studies. Risk ratios (RRs) and odds ratios (ORs) for each study calculated relative to youngest age group.
(DOCX)

Acknowledgments

LACC, ALKM, and TDH wish to thank Sarah Jervis and Paul Colognese for assistance with statistical analysis and programming, and Orin Courtenay and Erin Dilger of the University of Warwick for helpful discussions regarding visceral leishmaniasis diagnostics and their interpretation.

Author Contributions

Conceptualization: Lloyd A. C. Chapman, Caryn Bern, Graham F. Medley, T. Déirdre Hollingsworth.

Data curation: Lloyd A. C. Chapman, Alex L. K. Morgan.

Formal analysis: Lloyd A. C. Chapman, Alex L. K. Morgan, Graham F. Medley, T. Déirdre Hollingsworth.

Funding acquisition: T. Déirdre Hollingsworth.

Investigation: Lloyd A. C. Chapman.

Methodology: Lloyd A. C. Chapman, Caryn Bern, Graham F. Medley, T. Déirdre Hollingsworth.

Project administration: Lloyd A. C. Chapman, Graham F. Medley, T. Déirdre Hollingsworth.

Resources: T. Déirdre Hollingsworth.

Software: Lloyd A. C. Chapman, Graham F. Medley, T. Déirdre Hollingsworth.

Supervision: Emily R. Adams, Caryn Bern, Graham F. Medley, T. Déirdre Hollingsworth.

Validation: Lloyd A. C. Chapman.

Visualization: Lloyd A. C. Chapman, Caryn Bern, Graham F. Medley, T. Déirdre Hollingsworth.

Writing – original draft: Lloyd A. C. Chapman, Alex L. K. Morgan.

Writing – review & editing: Lloyd A. C. Chapman, Emily R. Adams, Caryn Bern, Graham F. Medley, T. Déirdre Hollingsworth.

References

1. Hirve S, Kroeger A, Matlashewski G, Mondal D, Banjara R, Das P, et al. Towards elimination of visceral leishmaniasis in the Indian subcontinent—Translating research to practice to public health. PLoS Negl Trop Dis. 2017; 11(10):e0005889. https://doi.org/10.1371/journal.pntd.0005889 PMID: 29023446

2. Le Rutte EA, Chapman LAC, Coffeng LE, Ruiz-Postigo JA, Olliaro PL, Adams ER, et al. Policy recommendations from transmission modelling for the elimination of visceral leishmaniasis in the Indian subcontinent. Clin Infect Dis. 2018;

3. Le Rutte EA, Chapman LAC, Coffeng LE, Jervis S, Hasker EC, Dwivedi S, et al. Elimination of visceral leishmaniasis in the Indian subcontinent: a comparison of predictions from three transmission models. Epidemics. 2017; 18:67–80. Available from: http://dx.doi.org/10.1016/j.epidem.2017.01.002 PMID: 28279458

4. Cameron MM, Acosta-Serrano A, Bern C, Boelaert M, den Boer M, Burza S, et al. Understanding the transmission dynamics of Leishmania donovani to provide robust evidence for interventions to eliminate visceral leishmaniasis in Bihar, India. Parasit Vectors. 2016; 9(1):25.

5. Drakeley CJ, Corran PH, Coleman PG, Tongren JE, McDonald SLR, Carneiro I, et al. Estimating medium- and long-term trends in malaria transmission by using serological markers of malaria exposure. Proc Natl Acad Sci. 2005; 102(14):5108–13. https://doi.org/10.1073/pnas.0408725102 PMID: 15792998

6. Cook J, Reid H, Iavro J, Kuwahata M, Taleo G, Clements A, et al. Using serological measures to monitor changes in malaria transmission in Vanuatu. Malar J. 2010; 9(1):1–15.

7. Pothin E, Ferguson NM, Drakeley CJ, Ghani AC. Estimating malaria transmission intensity from Plasmodium falciparum serological data using antibody density models. Malar J. 2016; 15(1):1–11.

8. Ferguson NM, Donnelly CA, Anderson RM. Transmission dynamics and epidemiology of dengue: insights from age-stratified sero-prevalence surveys. Philos Trans R Soc B Biol Sci. 1999; 354 (1384):757–68. Available from: http://rstb.royalsocietypublishing.org/cgi/doi/10.1098/rstb.1999.0428

9. Samuels AM, Clark EH, Galdos-Cardenas G, Wiegand RE, Ferrufino L, Menacho S, et al. Epidemiology of and impact of insecticide spraying on Chagas disease in communities in the Bolivian Chaco. PLoS Negl Trop Dis. 2013; 7(8):e2358. https://doi.org/10.1371/journal.pntd.0002358 PMID: 23936581

10. Delgado S, Castillo Neyra R, Quispe Machaca VR, Ancca Juárez J, Chou Chu L, Verastegui MR, et al. A History of Chagas Disease Transmission, Control, and Re-Emergence in Peri-Rural La Joya, Peru. Gürtler RE, editor. PLoS Negl Trop Dis. 2011; 5(2):e970. Available from: http://dx.plos.org/10.1371/journal.pntd.0000970 PMID: 21364970

11. Cucunubá ZM, Nouvellet P, Conteh L, Vera MJ, Angulo VM, Dib JC, et al. Modelling historical changes in the force-of-infection of Chagas disease to inform control and elimination programmes: application in Colombia. BMJ Glob Heal. 2017; 2(3):e000345. Available from: http://gh.bmj.com/lookup/doi/10.1136/bmjgh-2017-000345

12. Hollingsworth TD, Adams ER, Anderson RM, Atkins K, Bartsch S, Basáñez M, et al. Quantitative analyses and modelling to support achievement of the 2020 goals for nine neglected tropical diseases. Parasit Vectors. 2015; 8(1):630.

13. Rock KS, Le Rutte EA, de Vlas SJ, Adams ER, Medley GF, Hollingsworth TD. Uniting mathematics and biology for control of visceral leishmaniasis. Trends Parasitol. 2015; 31(6):251–9. Available from: http://linkinghub.elsevier.com/retrieve/pii/S1471492215000604 PMID: 25913079

14. Medley GF, Hollingsworth TD, Olliaro PL, Adams ER. Health-seeking behaviour, diagnostics and transmission dynamics in the control of visceral leishmaniasis in the Indian subcontinent. Nature. 2015; 528(7580):S102–8. Available from: http://www.nature.com/doifinder/10.1038/nature16042 PMID: 26633763

15. Biswas S, Subramanian A, Elmojtaba IM, Chattopadhyay J, Sarkar RR. Optimal combinations of control strategies and cost-effective analysis for visceral leishmaniasis disease transmission. PLoS One. 2017; 12(2):e0172465. https://doi.org/10.1371/journal.pone.0172465 PMID: 28222162

16. World Health Organization. Kala-azar elimination programme: Report of a WHO consultation of partners. World Health Organization;ISBN 978 92 4 150949 7. Geneva, Switzerland; 2015.

17. Special Programme for Research and Training in Tropical Diseases World Health Organization (WHO TDR). Eliminating visceral leishmaniasis: A multipronged approach. 2011 [cited 2017 Sep 17]. Available from: www.who.int/tdr/news/2011/vl-elimination/en/

18. Chapman L, Jewell CP, Spencer SEF, Pellis L, Datta S, Chowdhury R, et al. The role of case proximity in transmission of visceral leishmaniasis in a highly endemic village in Bangladesh. PLoS Negl Trop Dis. 2018; 12(10):e0006453. Available from: http://dx.doi.org/10.1371/journal.pntd.0006453 PMID: 30296295

19. Sen PK. VL elimination progress in India: where and when will the target be reached? SPEAK India general assembly meeting, Delhi 23–24 April, 2018. 2018 [cited 2018 Aug 1]. Available from: https://img1.wsimg.com/blobby/go/c5156b45-48df-4ba4-ab15-be2bb6261d20/downloads/1cdmv7qv5_7109.pdf

20. KalaCORE. Bangladesh. 2017 [cited 2018 Mar 7]. Available from: http://www.kalacore.org/where-we-work/bangladesh

21. Das P, Alvar J, Sharma B. Elimination of VL in the Indian subcontinent–is it achievable? 2014 [cited 2018 Mar 7]. Available from: http://www.dndi.org/wp-content/uploads/2014/07/Sharma_ICOPA_2014.pdf

22. Picado A, Singh SP, Rijal S, Sundar S, Ostyn B, Chappuis F, et al. Longlasting insecticidal nets for prevention of Leishmania donovani infection in India and Nepal: paired cluster randomised trial. BMJ. 2010; 341:c6760. https://doi.org/10.1136/bmj.c6760 PMID: 21190965

23. Hasker E, Kansal S, Malaviya P, Gidwani K, Picado A, Singh RP, et al. Latent infection with Leishmania donovani in highly endemic villages in Bihar, India. PLoS Negl Trop Dis. 2013; 7(2):e2053. https://doi.org/10.1371/journal.pntd.0002053 PMID: 23459501

24. Bern C, Haque R, Chowdhury R, Ali M, Kurkjian KM, Vaz L, et al. The epidemiology of visceral leishmaniasis and asymptomatic leishmanial infection in a highly endemic Bangladeshi village. Am J Trop Med Hyg. 2007; 76(5):909–14. PMID: 17488915

25. Bern C, Hightower AW, Chowdhury R, Ali M, Amann J, Wagatsuma Y, et al. Risk factors for kala-azar in Bangladesh. Emerg Infect Dis. 2005; 11(5):655–62. https://doi.org/10.3201/eid1105.040718 PMID: 15890115

26. Hasker E, Malaviya P, Gidwani K, Picado A, Ostyn B, Kansal S, et al. Strong association between serological status and probability of progression to clinical visceral leishmaniasis in prospective cohort studies in India and Nepal. PLoS Negl Trop Dis. 2014; 8(1):e2657. https://doi.org/10.1371/journal.pntd.0002657 PMID: 24466361

27. Hirve S, Boelaert M, Matlashewski G, Mondal D, Arana B, Kroeger A, et al. Transmission Dynamics of Visceral Leishmaniasis in the Indian Subcontinent–A Systematic Literature Review. PLoS Negl Trop Dis. 2016; 10(8):e0004896. Available from: http://dx.plos.org/10.1371/journal.pntd.0004896 PMID: 27490264

28. Sharma MC, Gupta a. K, Das VNR, Verma N, Kumar N, Saran R, et al. Leishmania donovani in blood smears of asymptomatic persons. Acta Trop. 2000; 76(2):195–6. PMID: 10936579

29. Das S, Matlashewski G, Bhunia GS, Kesari S, Das P. Asymptomatic Leishmania infections in northern India: a threat for the elimination programme? Trans R Soc Trop Med Hyg. 2014; 108(11):679–84. https://doi.org/10.1093/trstmh/tru146 PMID: 25205664

30. Singh OP, Hasker E, Sacks D, Boelaert M, Sundar S. Asymptomatic leishmania infection: A new challenge for leishmania control. Clin Infect Dis. 2014; 58(10):1424–9. https://doi.org/10.1093/cid/ciu102 PMID: 24585564

31. Rock KS, Quinnell RJ, Medley GF, Courtenay O. Progress in the Mathematical Modelling of Visceral Leishmaniasis. Vol. 94, Advances in Parasitology. Elsevier Ltd; 2016. 49–131 p. Available from: http://dx.doi.org/10.1016/bs.apar.2016.08.001

32. Heyneman D. Immunology of Leishmaniasis. Bull World Health Organ. 1971; 44:499–514. PMID: 5316252

33. Belehu A, Louis JA, Pugin P, Miescher PA. Immunopathological Aspects of Leishmaniasis. Springer Semin Immunopathol. 1980; 2:399–415.

34. Kumar R, Nylén S. Immunobiology of visceral leishmaniasis. Front Immunol. 2012; 3:1–10. https://doi.org/10.3389/fimmu.2012.00001

35. Kumar A. Leishmania and Leishmaniasis. Springer; 2013.

36. Neogy AB, Nandy A, Ghosh Dastidar B, Chowdhury AB. Leishmanin test in Indian kala-azar. Trans R Soc Trop Med Hyg. 1986; 80(3):454–5. PMID: 3798542

37. Nandy A, Neogy AB, Chowdhury AB. Leishmanin test survey in an endemic village of Indian kala-azar near Calcutta. Ann Trop Med Parasitol. 1987; 81(6):693–9. PMID: 3503646

38. Bern C, Amann J, Haque R, Chowdhury R, Ali M, Kurkjian KM, et al. Loss of leishmanin skin test antigen sensitivity and potency in a longitudinal study of visceral leishmaniasis in Bangladesh. Am J Trop Med Hyg. 2006; 75(4):744–8. PMID: 17038705

39. Khalil EAG, Zijlstra EE, Kager PA, Hassan AM El. Epidemiology and clinical manifestations of Leishmania donovani infection in two villages in an endemic area in eastern Sudan. Trop Med Int Heal. 2002; 7(1):35–44.

40. Manson-Bahr PE. Immunity in kala-azar. Trans R Soc Trop Med Hyg. 1961; 55(6):550–5. Available from: https://www.sciencedirect.com/science/article/pii/0035920361900785

41. Le Rutte EA, Coffeng LE, Bontje DM, Hasker EC, Postigo JAR, Argaw D, et al. Feasibility of eliminating visceral leishmaniasis from the Indian subcontinent: explorations with a set of deterministic age-structured transmission models. Parasit Vectors. 2016; 9:24. Available from: http://dx.doi.org/10.1186/s13071-016-1292-0 PMID: 26787302

42. Bollaerts K, Riera-Montes M, Heininger U, Hens N, Souverain A, Verstraeten T, et al. A systematic review of varicella seroprevalence in European countries before universal childhood immunization: deriving incidence from seroprevalence data. Epidemiol Infect. 2017; 145(13):2666–77. https://doi.org/10.1017/S0950268817001546 PMID: 28826422

43. Vynnycky E, Adams EJ, Cutts FT, Reef SE, Navar AM, Simons E, et al. Using seroprevalence and immunisation coverage data to estimate the global burden of Congenital Rubella Syndrome, 1996–2010: A systematic review. PLoS One. 2016; 11(3):e0149160. https://doi.org/10.1371/journal.pone.0149160 PMID: 26962867

44. Bern C, Courtenay O, Alvar J. Of Cattle, Sand Flies and Men: A Systematic Review of Risk Factor Analyses for South Asian Visceral Leishmaniasis and Implications for Elimination. PLoS Negl Trop Dis. 2010; 4(2):e599. https://doi.org/10.1371/journal.pntd.0000599 PMID: 20161727

45. Soni P, Mishra R, Kumar G, Pandey P, Kumar P. Kala-Azar Elimination in Bihar: Adoption of Newer Evidence-based Strategies Required for Elimination. Middle-East J Sci Res. 2013; 17(9):1220–7.

46. Guerin PJ, Olliaro P, Sundar S, Boelaert M, Croft SL, Desjeux P, et al. Visceral leishmaniasis: current status of control, diagnosis, and treatment, and a proposed research and development agenda. 2002; 2:494–501. PMID: 12150849

47. Wells GA, Shea B, O'Connell D, Peterson J, Welch V. Newcastle-Ottawa Scale (NOS) for assessing the quality of nonrandomised studies in meta-analyses. Available from: http://www.ohri.ca/programs/clinical_epidemiology/nosgen.pdf

48. Collaboration C. Part 2: General methods for Cochrane reviews. Cochrane Handb Syst Rev Interv. 2011;

49. Patil RR, Muliyil JP, Nandy A, Addy M, Maji A, Chatterjee P. Immuno-epidemiology of leishmanial infection among tribal population in kala-azar endemic areas: A community based study. Ann Trop Med Public Heal. 2013; 6(1):50. Available from: http://www.atmph.org/text.asp?2013/6/1/50/115193

50. Ostyn B, Gidwani K, Khanal B, Picado A, Chappuis FF, Singh SP, et al. Incidence of Symptomatic and Asymptomatic Leishmania donovani Infections in High-Endemic Foci in India and Nepal: A Prospective Study. PLoS Negl Trop Dis. 2011; 5(10):e1284. https://doi.org/10.1371/journal.pntd.0001284 PMID: 21991397

51. Chapman LAC, Dyson L, Courtenay O, Chowdhury R, Bern C, Medley GF, et al. Quantification of the natural history of visceral leishmaniasis and consequences for control. Parasit Vectors. 2015; 8 (1):521. Available from: http://www.parasitesandvectors.com/content/8/1/521

52. Bhattarai NR, Auwera G Van Der, Khanal B, Doncker S De, Rijal S, Boelaert M, et al. PCR and direct agglutination as Leishmania infection markers among healthy Nepalese subjects living in areas endemic for Kala-Azar. Trop Med Int Heal. 2009; 14(4):404–11.

53. Adams ER, Jacquet D, Schoone G, Gidwani K, Boelaert M. Leishmaniasis Direct Agglutination Test: Using Pictorials as Training Materials to Reduce Inter-Reader Variability and Improve Accuracy. PLoS Negl Trop Dis. 2012; 6(12):e1946. https://doi.org/10.1371/journal.pntd.0001946 PMID: 23272257

54. World Health Organization Regional Office for South-East Asia. Process of validation of elimination of kala-azar. 2016. Available from: http://www.who.int/leishmaniasis/resources/Process_of_validation_of_VL_elimination_SEA_CD_321.pdf?ua=1

55. Tummers B. DataThief III. 2006 [cited 2016 Jul 24]. Available from: http://datathief.org/

56. Lowry R. VassarStats: Website for Statistical Computation. 2016.

57. Cohen J. A Coefficient of Agreement for Nominal Scales. Educ Psychol Meas. 1960; 20(1):37–46. Available from: https://doi.org/10.1177/001316446002000104

58. Muench H. Catalytic Models in Epidemiology. Harvard University Press; 2014. Available from: https://www.degruyter.com/viewbooktoc/product/252696

59. Barnett PG, Singh SP, Bern C, Hightower AW, Sundar S. Virgin soil: the spread of visceral leishmaniasis into Uttar Pradesh, India. Am J Trop Med Hyg. 2005; 73(4):720–5. PMID: 16222016

60. Ferdousi F, Alam MS, Hossain MS, Ma E, Itoh M, Mondal D, et al. Visceral Leishmaniasis Eradication is a Reality: Data from a Community-based Active Surveillance in Bangladesh. Trop Med Health. 2012; 40(4):133–9. https://doi.org/10.2149/tmh.2012-25 PMID: 23532674

61. Hasker E, Singh SP, Malaviya P, Picado A, Gidwani K, Singh RP, et al. Visceral Leishmaniasis, Rural Bihar, India. Emerg Infect Dis. 2012; 18(10):1662–4. Available from: http://wwwnc.cdc.gov/eid/article/18/10/11-1083_article.htm PMID: 23017164

62. Picado A, Ostyn B, Singh SP, Uranw S, Hasker E, Rijal S, et al. Risk factors for visceral leishmaniasis and asymptomatic Leishmania donovani infection in India and Nepal. PLoS One. 2014; 9(1):e87641. https://doi.org/10.1371/journal.pone.0087641 PMID: 24498159

63. Singh VP, Ranjan A, Topno RK, Verma RB, Siddique NA, Ravidas VN, et al. Short Report: Estimation of Under-Reporting of Visceral Leishmaniasis Cases in Bihar, India. Am J Trop Med Hyg. 2010; 82 (1):9–11. Available from: http://www.ajtmh.org/cgi/doi/10.4269/ajtmh.2010.09-0235 PMID: 20064987

64. Kaushal H, Bhattacharya SK, Verma S, Salotra P. Serological and Molecular Analysis of Leishmania Infection in Healthy Individuals from Two Districts of West Bengal, India, Endemic for Visceral

Leishmaniasis. Am J Trop Med Hyg. 2017; 96(6):1448–55. https://doi.org/10.4269/ajtmh.16-0592 PMID: 28719266

65. Koirala S, Karki P, Das ML, Parija SC, Karki BMS. Epidemiological study of kala-azar by direct agglutination test in two rural communities of eastern Nepal. Trop Med Int Heal. 2004; 9(4):533–7.

66. Ostyn B, Uranw S, Bhattarai NR, Das ML, Rai K, Tersago K, et al. Transmission of Leishmania donovani in the Hills of Eastern Nepal, an Outbreak Investigation in Okhaldhunga and Bhojpur Districts. PLoS Negl Trop Dis. 2015; 9(8):e0003966. Available from: http://dx.plos.org/10.1371/journal.pntd.0003966 PMID: 26252494

67. Rijal S, Uranw S, Chappuis F, Picado A, Khanal B, Paudel IS, et al. Epidemiology of Leishmania donovani infection in high-transmission foci in Nepal. Trop Med Int Heal. 2010; 15(Suppl. 2):21–8.

68. Schenkel K, Rijal S, Koirala SS, Koirala SS, Vanlerberghe V, Van der Stuyft P, et al. Visceral leishmaniasis in southeastern Nepal: A cross-sectional survey on Leishmania donovani infection and its risk factors. Trop Med Int Heal. 2006; 11(12):1792–9.

69. Singh SP, Picado A, Boelaert M, Gidwani K, Andersen EW, Ostyn B, et al. The epidemiology of Leishmania donovani infection in high transmission foci in India. Trop Med Int Heal. 2010; 15(Suppl. 2):12–20.

70. Topno RK, Das VNR, Ranjan A, Pandey K, Singh D, Kumar N, et al. Asymptomatic infection with visceral leishmaniasis in a disease-endemic area in Bihar, India. Am J Trop Med Hyg. 2010; 83(3):502–6. https://doi.org/10.4269/ajtmh.2010.09-0345 PMID: 20810810

71. Yangzom T, Cruz I, Bern C, Argaw D, den Boer M, Vélez ID, et al. Endemic transmission of visceral leishmaniasis in Bhutan. Am J Trop Med Hyg. 2012; 87(6):1028–37. https://doi.org/10.4269/ajtmh.2012.12-0211 PMID: 23091191

72. Kurkjian KM, Vaz LE, Haque R, Cetre-Sossah C, Akhter S, Roy S, et al. Application of an Improved Method for the Recombinant K39 Enzyme-Linked Immunosorbent Assay To Detect Visceral Leishmaniasis Disease and Infection in Bangladesh. Clin Diagn Lab Immunol. 2005; 12(12):1410–5. https://doi.org/10.1128/CDLI.12.12.1410-1415.2005 PMID: 16339064

73. Matlashewski G, Das VNR, Pandey K, Singh D, Das S, Ghosh AK, et al. Diagnosis of visceral leishmaniasis in Bihar India: comparison of the rK39 rapid diagnostic test on whole blood versus serum. PLoS Negl Trop Dis. 2013; 7(5):e2233. https://doi.org/10.1371/journal.pntd.0002233 PMID: 23717700

74. Landis JR, Koch GG. The Measurement of Observer Agreement for Categorical Data. Biometrics. 1977; 33(1):159–74. Available from: http://www.jstor.org/stable/2529310 PMID: 843571

75. Gidwani K, Picado A, Ostyn B, Singh SP, Kumar R, Khanal B, et al. Persistence of Leishmania donovani Antibodies in Past Visceral Leishmaniasis Cases in India. Clin Vaccine Immunol. 2011; 18 (2):346–8. https://doi.org/10.1128/CVI.00473-10 PMID: 21159922

76. World Health Organization. Global Health Observatory: Number of cases of visceral leishmaniasis reported—Data by country. World Health Organization; 2017 [cited 2017 Aug 23]. Available from: http://apps.who.int/gho/data/node.main.NTDLEISHVNUM?lang=en

77. Jervis S, Chapman LAC, Dwivedi S, Karthick M, Das A, Le Rutte EA, et al. Variations in visceral leishmaniasis burden, mortality and the pathway to care within Bihar, India. Parasit Vectors. 2017; 10 (1):601. https://doi.org/10.1186/s13071-017-2530-9 PMID: 29216905

78. Hailu A, Gramiccia M, Kager PA, Immunomediate P, Superiore I, Elena VR. Visceral leishmaniasis in Aba-Roba, south-western Ethiopia: prevalence and incidence of active and subclinical infections. Ann Trop Med Parasitol. 2009; 103(8):659–70. https://doi.org/10.1179/000349809X12554106963555 PMID: 20030990

79. Gidwani K, Rai M, Chakravarty J, Boelaert M, Sundar S. Short Report: Evaluation of leishmanin skin test in Indian visceral leishmaniasis. Am J Trop Med Hyg. 2009; 80(4):566–7. PMID: 19346376

80. Dye C, Wolpert DM. Earthquakes, influenza and cycles of Indian kala-azar. Trans R Soc Trop Med Hyg. 1988; 82(6):843–50. PMID: 3256984

81. Bora D. Epidemiology of visceral leishmaniasis in India. Natl Med J India. 1999; 12(2):62–8. PMID: 10416321

82. Courtenay O, Peters NC, Rogers ME, Bern C. Combining epidemiology with basic biology of sand flies, parasites and hosts to inform leishmaniasis transmission dynamics and control. PLoS Pathog. 2017; 13(10):e1006571. https://doi.org/10.1371/journal.ppat.1006571 PMID: 29049371

83. Picado A, Ostyn B, Rijal S, Sundar S, Singh SP, Chappuis F, et al. Long-lasting Insecticidal Nets to Prevent Visceral Leishmaniasis in the Indian Subcontinent; Methodological Lessons Learned from a Cluster Randomised Controlled Trial. PLoS Negl Trop Dis. 2015; 9(4):e0003597. https://doi.org/10.1371/journal.pntd.0003597 PMID: 25856238

84. Malaviya P, Picado A, Singh SP, Hasker E, Singh RP, Sundar S, et al. Visceral Leishmaniasis in Muzaffarpur District, Bihar, India from 1990 to 2008. PLoS One. 2011; 6(3):e14751. https://doi.org/10.1371/journal.pone.0014751 PMID: 21394195

85. Akter S, Alam MZ, Islam MT, Mondal MMH. Seroepidemiological study of visceral leishmaniasis and cattle as a possible reservoir host at Trishal Upazila in Bangladesh. J Bangladesh Agric Univ. 2012; 10(1):79–86.

86. Ali A, Ashford RW. Visceral leishmaniasis in Ethiopia. II. Annual leishmanin transformation in a population. Is positive leishmanin reaction a life-long phenomenon? Ann Trop Med Parasitol. 1993; 87(2):163–7. PMID: 8561523

87. Gidwani K, Kumar R, Rai M, Sundar S. Longitudinal Seroepidemiologic Study of Visceral Leishmaniasis in Hyperendemic Regions of Bihar, India. Am J Trop Med Hyg. 2009; 80(3):345–6. PMID: 19270279

88. Khanal B, Rijal S, Ostyn B, Picado A, Gidwani K, Menten J, et al. Serological markers for Leishmania donovani infection in Nepal: Agreement between direct agglutination test and rK39 ELISA. Trop Med Int Heal. 2010; 15(11):1390–4.

89. Sudarshan M, Singh T, Singh AK, Chourasia A, Singh B, Wilson ME, et al. Quantitative PCR in Epidemiology for Early Detection of Visceral Leishmaniasis Cases in India. PLoS Negl Trop Dis. 2014; 8(12):e3366. https://doi.org/10.1371/journal.pntd.0003366 PMID: 25503103

90. Stauch A, Sarkar RR, Picado A, Ostyn B, Sundar S, Rijal S, et al. Visceral leishmaniasis in the Indian subcontinent: modelling epidemiology and control. PLoS Negl Trop Dis. 2011; 5(11):e1405. https://doi.org/10.1371/journal.pntd.0001405 PMID: 22140589

91. Patil R, Muliyil J, Nandy A, Addy A, Maji AK, Chatterjee P. Dynamics of the antibodies in cohorts of cured cases of visceral leishmaniasis: Its implication on the validity of serological test, value in prognosis and in post therapeutic assessment. Hum Vaccin Immunother. 2012; 8(6):725–30. Available from: http://www.tandfonline.com/doi/abs/10.4161/auto.8.1.18339 PMID: 22495122

92. Sudarshan M, Weirather JL, Wilson ME, Sundar S. Study of parasite kinetics with antileishmanial drugs using real-time quantitative PCR in Indian visceral leishmaniasis. J Antimicrob Chemother. 2011; 66:1751–5. https://doi.org/10.1093/jac/dkr185 PMID: 21609983

93. Menten J, Boelaert M, Lesaffre E. An application of Bayesian growth mixture modelling to estimate infection incidences from repeated serological tests. Stat Modelling. 2012; 12(6):551–78.

94. Perry D, Dixon K, Garlapati R, Gendernalik A, Poche D, Poche R, et al. Visceral leishmaniasis prevalence and associated risk factors in the Saran District of Bihar, India, from 2009 to July of 2011. Am J Trop Med Hyg. 2013; 88(4):778–84. https://doi.org/10.4269/ajtmh.12-0442 PMID: 23382167

95. Burza S, Sinha PK, Mahajan R, Lima MA, Mitra G, Verma N, et al. Five-Year Field Results and Long-Term Effectiveness of 20 mg/kg Liposomal Amphotericin B (Ambisome) for Visceral Leishmaniasis in Bihar, India. PLoS Negl Trop Dis. 2014; 8(1):e2603. https://doi.org/10.1371/journal.pntd.0002603 PMID: 24392168

96. Bern C, Maguire JH, Alvar J. Complexities of Assessing the Disease Burden Attributable to Leishmaniasis. PLoS Negl Trop Dis. 2008; 2(10):e313. Available from: http://dx.plos.org/10.1371/journal.pntd.0000313 PMID: 18958165

97. Snider H, Lezama-Davila C, Alexander J, Satoskar AR. Sex Hormones and Modulation of Immunity against Leishmaniasis. Neuroimmunomodulation. 2009; 16(2):106–13. https://doi.org/10.1159/000180265 PMID: 19212130

98. Ahluwalia IB, Bern C, Costa C, Akter T, Chowdhury R, Ali M, et al. Visceral leishmaniasis: Consequences of a neglected disease in a Bangladeshi community. Am J Trop Med Hyg. 2003; 69(6):624–8. PMID: 14740879

99. Hailu A, Skrip L, Kirstein O, Shiferaw W, Belay S, Aysheshm K, et al. Associations between parasitological and serological indicators of infection and the development of clinical Visceral Leishmaniasis in Ethiopia. 2016 [cited 2018 Mar 7]. Available from: http://www.abstractsonline.com/pp8/#!/4114/presentation/1289

100. Singh OP, Sundar S. Developments in diagnosis of visceral leishmaniasis in the elimination era. J Parasitol Res. 2015;2015:1–10. Available from: http://www.hindawi.com/journals/jpr/2015/239469/

101. Ghosh P, Bhaskar KRH, Hossain F, Khan MAA, Vallur AC, Duthie MS, et al. Evaluation of diagnostic performance of rK28 ELISA using urine for diagnosis of visceral leishmaniasis. Parasit Vectors. 2016; 9(1):383. Available from: http://parasitesandvectors.biomedcentral.com/articles/10.1186/s13071-016-1667-2 PMID: 27377266
